# Supplementary material for: Comparative effectiveness over time of the mRNA-1273 (Moderna) vaccine and the BNT162b2 (Pfizer-BioNTech) vaccine
Source: Nat Commun. 2022 May 2;13:2377. doi: 10.1038/s41467-022-30059-3 (PMC9061778; doi:10.1038/s41467-022-30059-3)
Supplement: Supplementary file 1 — Supplementary Information [file 41467_2022_30059_MOESM1_ESM.pdf]

SUPPLEMENTAL MATERIAL FOR

Comparative Effectiveness over time of the mRNA-1273 (Moderna) vaccine and the BNT162b2 (Pfizer-BioNTech) vaccine

Nazmul Islam, PhD, MBA
Natalie E. Sheils, PhD
Megan S. Jarvis, MS
Kenneth Cohen, MD

Table S1. Descriptive statistics for fully vaccinated individuals.

Descriptive statistics for baseline characteristics (demographics, socio-economic, comorbidities, negative controls, study outcomes, and other prognostic factors) among fully vaccinated individuals by BNT162b2 and mRNA-1273. Analytical datasets are separated into those fully vaccinated by May 1, 2021, April 1, 2021, and March 2, 2021, ensuring each patient had at least 30, 60, and 90 days of post-vaccination follow-up to experience events, respectively. For time-to-event outcomes we consider individuals vaccinated by June 1, 2021.

	30 DAY POST-VACCINATION BINARY OUTCOME		60 DAY POST-VACCINATION BINARY OUTCOME		90 DAY POST-VACCINATION BINARY OUTCOME		POST-VACCINATION TIME-TO-EVENT	
	BNT162B2	MRNA-1273	BNT162B2	MRNA-1273	BNT162B2	MRNA-1273	BNT162B2	MRNA-1273
N	1,380,571	1,058,703	577,390	576,021	218,727	168,203	2,364,605	1,598,450
Age, mean (SD)	56.17 (17.30)	59.82 (17.42)	61.14 (18.25)	62.62 (17.67)	57.16 (19.30)	56.14 (18.34)	50.67 (17.46)	55.39 (17.84)
18–24, n (%)	52,066 (3.8)	29,405 (2.8)	15,358 (2.7)	13,165 (2.3)	7,808 (3.6)	5,891 (3.5)	168,893 (7.1)	71,792 (4.5)
25–29, n (%)	58,013 (4.2)	37,442 (3.5)	21,091 (3.7)	18,745 (3.3)	11,076 (5.1)	8,758 (5.2)	147,969 (6.3)	79,056 (4.9)
30–34, n (%)	76,583 (5.5)	47,585 (4.5)	26,230 (4.5)	22,788 (4.0)	13,865 (6.3)	10,723 (6.4)	183,193 (7.7)	98,126 (6.1)
35–39, n (%)	89,903 (6.5)	56,971 (5.4)	30,647 (5.3)	27,038 (4.7)	15,720 (7.2)	12,506 (7.4)	200,901 (8.5)	111,811 (7.0)
40–44, n (%)	96,788 (7.0)	61,615 (5.8)	32,872 (5.7)	28,914 (5.0)	16,282 (7.4)	12,757 (7.6)	205,561 (8.7)	116,946 (7.3)
45–49, n (%)	99,700 (7.2)	62,452 (5.9)	33,286 (5.8)	29,642 (5.1)	16,445 (7.5)	12,955 (7.7)	204,181 (8.6)	115,928 (7.3)
50–54, n (%)	121,340 (8.8)	73,665 (7.0)	37,000 (6.4)	33,312 (5.8)	17,623 (8.1)	14,084 (8.4)	229,689 (9.7)	133,745 (8.4)
55–59, n (%)	143,418 (10.4)	86,363 (8.2)	39,811 (6.9)	35,042 (6.1)	18,449 (8.4)	13,867 (8.2)	243,091 (10.3)	149,095 (9.3)
60–64, n (%)	167,142 (12.1)	106,160 (10.0)	42,755 (7.4)	38,359 (6.7)	17,687 (8.1)	13,688 (8.1)	251,989 (10.7)	167,022 (10.4)
65–69, n (%)	143,639 (10.4)	135,179 (12.8)	68,104 (11.8)	75,536 (13.1)	16,323 (7.5)	15,189 (9.0)	169,161 (7.2)	159,330 (10.0)
70–74, n (%)	129,062 (9.3)	140,307 (13.3)	80,180 (13.9)	93,025 (16.1)	18,926 (8.7)	17,087 (10.2)	141,787 (6.0)	154,884 (9.7)
75–79, n (%)	92,331 (6.7)	104,571 (9.9)	65,726 (11.4)	74,742 (13.0)	17,706 (8.1)	13,653 (8.1)	99,802 (4.2)	113,859 (7.1)
80–84, n (%)	56,716 (4.1)	63,067 (6.0)	41,957 (7.3)	45,913 (8.0)	13,415 (6.1)	8,911 (5.3)	60,991 (2.6)	68,607 (4.3)
85+, n (%)	53,870 (3.9)	53,921 (5.1)	42,373 (7.3)	39,800 (6.9)	17,402 (8.0)	8,134 (4.8)	57,397 (2.4)	58,249 (3.6)
Insurance type, n (%)								
Medicare advantage	362,457 (26.3)	405,279 (38.3)	233,910 (40.5)	271,806 (47.2)	67,488 (30.9)	51,398 (30.6)	406,023 (17.2)	460,303 (28.8)
Commercial	1,018,114 (73.7)	653,424 (61.7)	343,480 (59.5)	304,215 (52.8)	151,239 (69.1)	116,805 (69.4)	1,958,582 (82.8)	1,138,147 (71.2)
Acquired immune deficiency syndrome (%)	4,101 (0.3)	2,674 (0.3)	1,385 (0.2)	1,219 (0.2)	505 (0.2)	349 (0.2)	5,979 (0.3)	4,089 (0.3)
Alcohol use disorder (%)	13,744 (1.0)	10,686 (1.0)	5,887 (1.0)	5,462 (0.9)	2,276 (1.0)	1,296 (0.8)	23,067 (1.0)	16,505 (1.0)
Iron deficiency anemia (%)	99,142 (7.2)	88,855 (8.4)	53,919 (9.3)	54,164 (9.4)	20,483 (9.4)	13,470 (8.0)	134,449 (5.7)	113,554 (7.1)
Rheumatoid	50,701 (3.7)	44,456 (4.2)	24,377 (4.2)	26,146 (4.5)	7,877 (3.6)	6,241 (3.7)	68,719 (2.9)	57,650 (3.6)

arthritis (%)								
Blood loss anemia (%)	10,920 (0.8)	8,887 (0.8)	5,473 (0.9)	5,192 (0.9)	2,056 (0.9)	1,352 (0.8)	16,658 (0.7)	12,168 (0.8)
Congestive heart failure (%)	46,021 (3.3)	44,151 (4.2)	28,835 (5.0)	28,344 (4.9)	11,433 (5.2)	6,080 (3.6)	54,459 (2.3)	52,766 (3.3)
Chronic obstructive pulmonary disease (%)	131,516 (9.5)	117,089 (11.1)	65,241 (11.3)	68,291 (11.9)	23,419 (10.7)	16,706 (9.9)	181,928 (7.7)	153,476 (9.6)
Coagulopathy (%)	20,653 (1.5)	18,236 (1.7)	11,342 (2.0)	11,228 (1.9)	3,922 (1.8)	2,541 (1.5)	27,144 (1.1)	22,779 (1.4)
Depression (%)	137,738 (10.0)	106,496 (10.1)	64,167 (11.1)	59,532 (10.3)	26,872 (12.3)	16,688 (9.9)	220,368 (9.3)	156,024 (9.8)
Diabetes without chronic complication (%)	168,834 (12.2)	156,846 (14.8)	82,758 (14.3)	90,877 (15.8)	27,313 (12.5)	20,735 (12.3)	220,001 (9.3)	199,263 (12.5)
Diabetes with chronic complication (%)	128,771 (9.3)	122,046 (11.5)	65,518 (11.3)	71,168 (12.4)	21,289 (9.7)	15,606 (9.3)	165,082 (7.0)	153,820 (9.6)
Substance use disorder (%)	13,877 (1.0)	11,199 (1.1)	7,219 (1.3)	6,159 (1.1)	3,544 (1.6)	1,593 (0.9)	21,148 (0.9)	16,534 (1.0)
Hypertension (%)	492,622 (35.7)	444,784 (42.0)	246,757 (42.7)	264,127 (45.9)	81,049 (37.1)	60,709 (36.1)	650,386 (27.5)	565,890 (35.4)
Hypothyroidism (%)	158,162 (11.5)	137,192 (13.0)	79,897 (13.8)	82,745 (14.4)	28,248 (12.9)	20,765 (12.3)	216,239 (9.1)	176,485 (11.0)
Liver disease (%)	47,320 (3.4)	38,487 (3.6)	20,538 (3.6)	21,050 (3.7)	6,690 (3.1)	5,202 (3.1)	68,693 (2.9)	52,833 (3.3)
Lymphoma (%)	7,408 (0.5)	6,127 (0.6)	4,023 (0.7)	3,878 (0.7)	1,125 (0.5)	813 (0.5)	9,198 (0.4)	7,294 (0.5)
Fluid & electrolyte disorder (%)	63,774 (4.6)	56,155 (5.3)	35,383 (6.1)	34,021 (5.9)	13,612 (6.2)	7,955 (4.7)	83,834 (3.5)	71,552 (4.5)
Metastatic cancer (%)	11,392 (0.8)	8,884 (0.8)	5,732 (1.0)	5,411 (0.9)	1,612 (0.7)	1,142 (0.7)	14,154 (0.6)	10,789 (0.7)
Neurological disorder (%)	69,786 (5.1)	58,082 (5.5)	41,046 (7.1)	35,846 (6.2)	17,898 (8.2)	8,672 (5.2)	90,589 (3.8)	73,480 (4.6)
Obesity (%)	190,934 (13.8)	160,424 (15.2)	79,747 (13.8)	87,058 (15.1)	27,858 (12.7)	23,311 (13.9)	285,300 (12.1)	224,163 (14.0)
Paralysis (%)	10,351 (0.7)	8,130 (0.8)	6,680 (1.2)	4,987 (0.9)	3,679 (1.7)	1,389 (0.8)	12,828 (0.5)	10,341 (0.6)
Peripheral vascular disease (%)	83,258 (6.0)	80,557 (7.6)	53,983 (9.3)	52,985 (9.2)	20,143 (9.2)	11,115 (6.6)	96,871 (4.1)	94,013 (5.9)
Psychosis (%)	41,072 (3.0)	30,690 (2.9)	19,741 (3.4)	17,113 (3.0)	8,821 (4.0)	4,702 (2.8)	64,967 (2.7)	45,815 (2.9)
Pulmonary circulation disorder (%)	9,297 (0.7)	7,811 (0.7)	4,987 (0.9)	4,628 (0.8)	1,726 (0.8)	1,022 (0.6)	11,977 (0.5)	9,880 (0.6)
Chronic kidney disease (%)	76,924 (5.6)	78,401 (7.4)	48,601 (8.4)	51,012 (8.9)	16,182 (7.4)	10,209 (6.1)	89,976 (3.8)	91,653 (5.7)

Solid tumor without metastasis (%)	76,075 (5.5)	64,931 (6.1)	40,037 (6.9)	40,412 (7.0)	11,871 (5.4)	8,749 (5.2)	94,664 (4.0)	78,162 (4.9)
Peptic ulcer disease (%)	6,257 (0.5)	5,554 (0.5)	3,194 (0.6)	3,346 (0.6)	1,084 (0.5)	756 (0.4)	8,540 (0.4)	7,202 (0.5)
Valvular disorder (%)	65,913 (4.8)	62,905 (5.9)	37,986 (6.6)	40,262 (7.0)	12,237 (5.6)	9,176 (5.5)	80,847 (3.4)	74,869 (4.7)
Weight loss (%)	25,374 (1.8)	20,193 (1.9)	15,266 (2.6)	12547 (2.2)	6,986 (3.2)	3,084 (1.8)	34,102 (1.4)	25,769 (1.6)
Stroke cerebrovascular (%)	74,486 (5.4)	70,488 (6.7)	45,373 (7.9)	45248 (7.9)	16,311 (7.5)	10,164 (6.0)	89,341 (3.8)	83,594 (5.2)
Down syndrome (%)	316 (0.0)	252 (0.0)	120 (0.0)	123 (0.0)	52 (0.0)	28 (0.0)	419 (0.0)	329 (0.0)
Thalassemia (%)	1,608 (0.1)	1,198 (0.1)	670 (0.1)	669 (0.1)	235 (0.1)	181 (0.1)	2,510 (0.1)	1,659 (0.1)
Smoking (%)	64,362 (4.7)	56,446 (5.3)	28,385 (4.9)	30872 (5.4)	9,589 (4.4)	7,521 (4.5)	96,870 (4.1)	79,584 (5.0)
Transplant (%)	1,127 (0.1)	909 (0.1)	521 (0.1)	521 (0.1)	127 (0.1)	116 (0.1)	1,486 (0.1)	1,115 (0.1)
Elixhauser mortality score, mean (SD)	1.65 (4.75)	1.90 (5.01)	2.27 (5.64)	2.19 (5.36)	2.22 (5.78)	1.67 (4.74)	1.23 (4.08)	1.56 (4.55)
Elixhauser readmission score, mean (SD)	5.95 (11.64)	6.87 (12.22)	7.76 (13.66)	7.63 (12.86)	7.68 (14.59)	6.05 (11.76)	4.65 (10.16)	5.83 (11.32)
Transferred from nursing facility/ SNF (%)	2,368 (0.2)	719 (0.1)	2,236 (0.4)	609 (0.1)	1,873 (0.9)	420 (0.2)	2,465 (0.1)	832 (0.1)
Immunologic Rx (%)	339,719 (24.6)	297,923 (28.1)	165,840 (28.7)	177,605 (30.8)	59,176 (27.1)	45,565 (27.1)	496,795 (21.0)	397,420 (24.9)
Immunologic Dx (%)	390,067 (28.3)	318,405 (30.1)	183,400 (31.8)	188,610 (32.7)	60,008 (27.4)	47,794 (28.4)	556,640 (23.5)	420,524 (26.3)
Sex								
Female (%)	780,162 (56.5)	608,628 (57.5)	354,134 (61.3)	344,902 (59.9)	143,348 (65.5)	105,587 (62.8)	1,264,075 (53.5)	874,407 (54.7)
Male (%)	600,409 (43.5)	450,075 (42.5)	223,256 (38.7)	231,119 (40.1)	75,379 (34.5)	62,616 (37.2)	1,100,530 (46.5)	724,043 (45.3)
Residence by region (%)								
Midwest	439,164 (31.8)	304,757 (28.8)	185,910 (32.2)	164,256 (28.5)	67,205 (30.7)	39,086 (23.2)	752,567 (31.8)	467,702 (29.3)
Northeast	190,544 (13.8)	155,541 (14.7)	77,097 (13.4)	80,167 (13.9)	33,699 (15.4)	32,208 (19.1)	335,877 (14.2)	228,427 (14.3)
South	395,294 (28.6)	323,742 (30.6)	141,732 (24.5)	172,565 (30.0)	58,464 (26.7)	64,696 (38.5)	672,676 (28.4)	481,238 (30.1)
West	355,569 (25.8)	274,663 (25.9)	172,651 (29.9)	159,033 (27.6)	59,359 (27.1)	32,213 (19.2)	60,3485 (25.5)	421,083 (26.3)
Residence class (%)								
Rural	208,196 (15.1)	278,152 (26.3)	99,749 (17.3)	160,591 (27.9)	44,281 (20.2)	45,163 (26.9)	315,989 (13.4)	384,932 (24.1)
Urban	590,527 (42.8)	365,864 (34.6)	241,579 (41.8)	195,154 (33.9)	87,062 (39.8)	57,818 (34.4)	1,033,537 (43.7)	576,903 (36.1)

Semi-urban	581,848 (42.1)	414,687 (39.2)	236,062 (40.9)	220,276 (38.2)	87,384 (40.0)	65,222 (38.8)	1,015,079 (42.9)	636,615 (39.8)
First dose administered before Feb 1, 2021* (%)	317,863 (23.0)	359,372 (33.9)	317,863 (55.1)	359,372 (62.4)	86,899 (39.7)	82,298 (48.9)	317,863 (13.4)	359,372 (22.5)
SES index, mean (SD)	53.36 (2.85)	52.85 (3.13)	53.12 (2.80)	52.68 (3.15)	52.91 (2.80)	52.81 (3.34)	53.70 (2.90)	53.08 (3.12)
Prior any Covid dx (ER/IP/ICU) (%)	99,011 (7.2)	67,839 (6.4)	44,286 (7.7)	34,856 (6.1)	21,623 (9.9)	11,740 (7.0)	169,077 (7.2)	107,891 (6.7)
Prior positive PCR (%)	21,496 (1.6)	15,169 (1.4)	8,070 (1.4)	7,319 (1.3)	3,386 (1.5)	2,361 (1.4)	40,055 (1.7)	25,595 (1.6)
Negative outcomes, N (%)								
Bilateral prim osteoarthritis knee	19,543 (1.4)	17,431 (1.6)	10,174 (1.8)	10,692 (1.9)	3,157 (1.4)	2,324 (1.4)	24,782 (1.0)	21,626 (1.4)
Dry eye syndrome	2 (0.0)	1 (0.0)	0 (0.0)	1 (0.0)	0 (0.0)	0 (0.0)	3 (0.0)	2 (0.0)
Frequency of micturition	31,803 (2.3)	26,551 (2.5)	16,187 (2.8)	16,091 (2.8)	5,538 (2.5)	3,954 (2.4)	44,767 (1.9)	34,724 (2.2)
Presbyopia	38,211 (2.8)	35,425 (3.3)	20,012 (3.5)	21,946 (3.8)	6,212 (2.8)	4,673 (2.8)	48,763 (2.1)	43,324 (2.7)
Seborrheic keratosis	0 (0.0)	0 (0.0)	0 (0.0)	0 (0.0)	0 (0.0)	0 (0.0)	0 (0.0)	0 (0.0)
Sensorineural hear loss bilateral	25,002 (1.8)	22,245 (2.1)	14,574 (2.5)	14,694 (2.6)	4,700 (2.1)	3,435 (2.0)	30,894 (1.3)	26,304 (1.6)
Tinea unguium	44,379 (3.2)	37,040 (3.5)	29,098 (5.0)	24,299 (4.2)	13,241 (6.1)	5,980 (3.6)	54,331 (2.3)	44,673 (2.8)
UTI site not specified	61,669 (4.5)	54,508 (5.1)	34,683 (6.0)	33,859 (5.9)	14,563 (6.7)	9,470 (5.6)	84,857 (3.6)	70,241 (4.4)
Agerel nuclear cataract bilateral	65,033 (4.7)	62,082 (5.9)	35,631 (6.2)	39,377 (6.8)	10,195 (4.7)	8,690 (5.2)	78,050 (3.3)	72,712 (4.5)
Study-related outcomes								
Infection (%)	3,766 (0.3)	1,949 (0.2)	3,376 (0.6)	1,724 (0.3)	2,514 (1.1)	861 (0.5)	5,758 (0.2)	3,090 (0.2)
Hospitalization/ ICU/ deceased/ transferred to hospice (%)	72 (0.0)	42 (0.0)	82 (0.0)	49 (0.0)	55 (0.0)	23 (0.0)	129 (0.0)	85 (0.0)
ICU/ deceased/ transferred to hospice (%)	32 (0.0)	14 (0.0)	36 (0.0)	21 (0.0)	26 (0.0)	8 (0.0)	57 (0.0)	40 (0.0)
Hospitalization (%)	58 (0.0)	35 (0.0)	67 (0.0)	39 (0.0)	46 (0.0)	21 (0.0)	105 (0.0)	69 (0.0)
ICU (%)	28 (0.0)	14 (0.0)	30 (0.0)	21 (0.0)	20 (0.0)	7 (0.0)	47 (0.0)	38 (0.0)

Deceased/ transferred to hospice (%)	9 (0.0)	2 (0.0)	12 (0.0)	1 (0.0)	10 (0.0)	1 (0.0)	21 (0.0)	7 (0.0)

*For the 90-day outcome the date was adjusted to “First dose administered before January 10, 2021.”

Figure S1. Occupation probabilities of different states over time.
Differences between first and second dose for (A) BNT-162b2 and (B) mRNA-1273. Dotted lines correspond to proportions shown above at days 50 and 150 for each population.

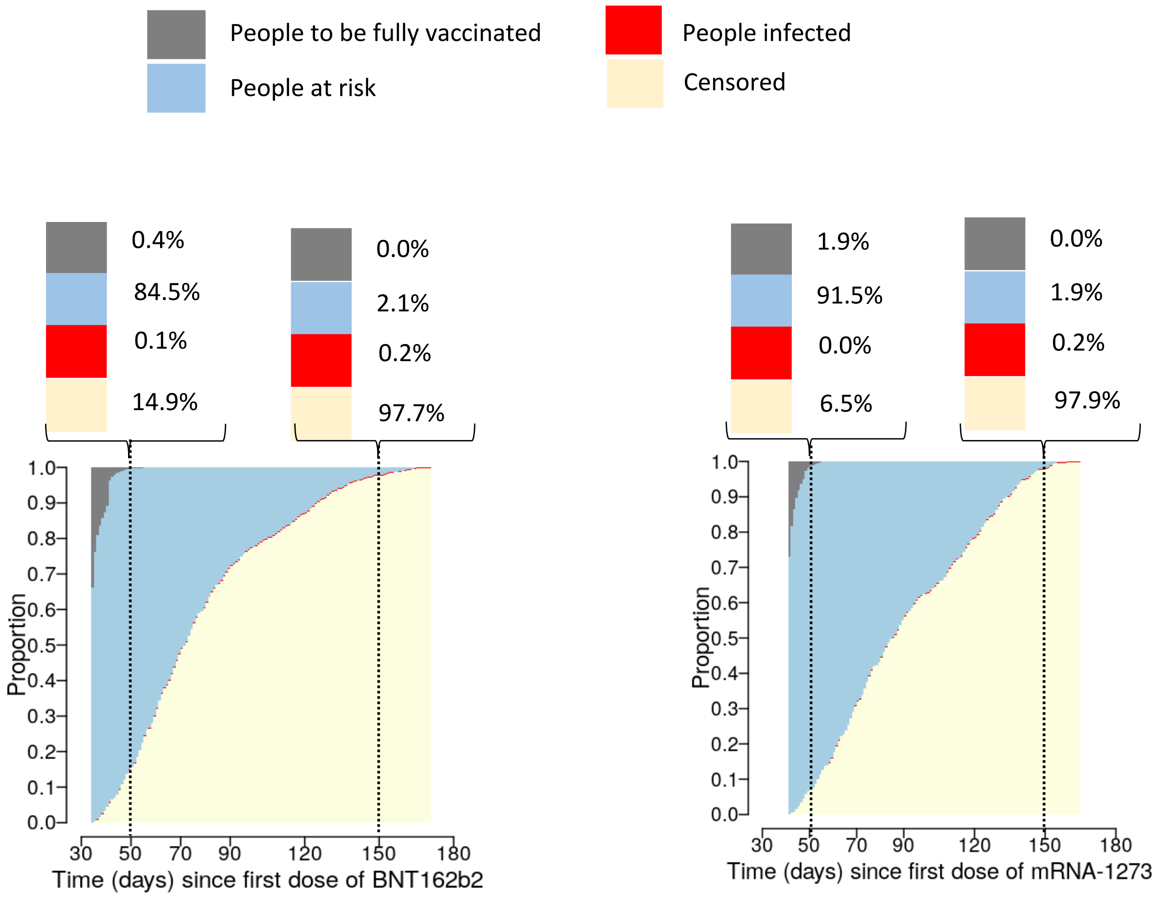

(A) (B)

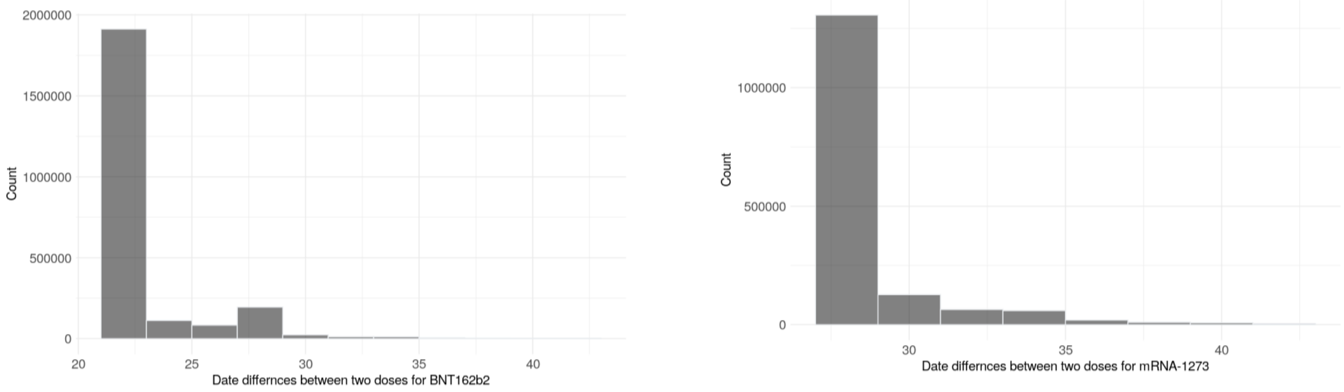

Figure S2. Distribution of first doses by calendar date corresponding to mRNA-1273 and BNT162b2.

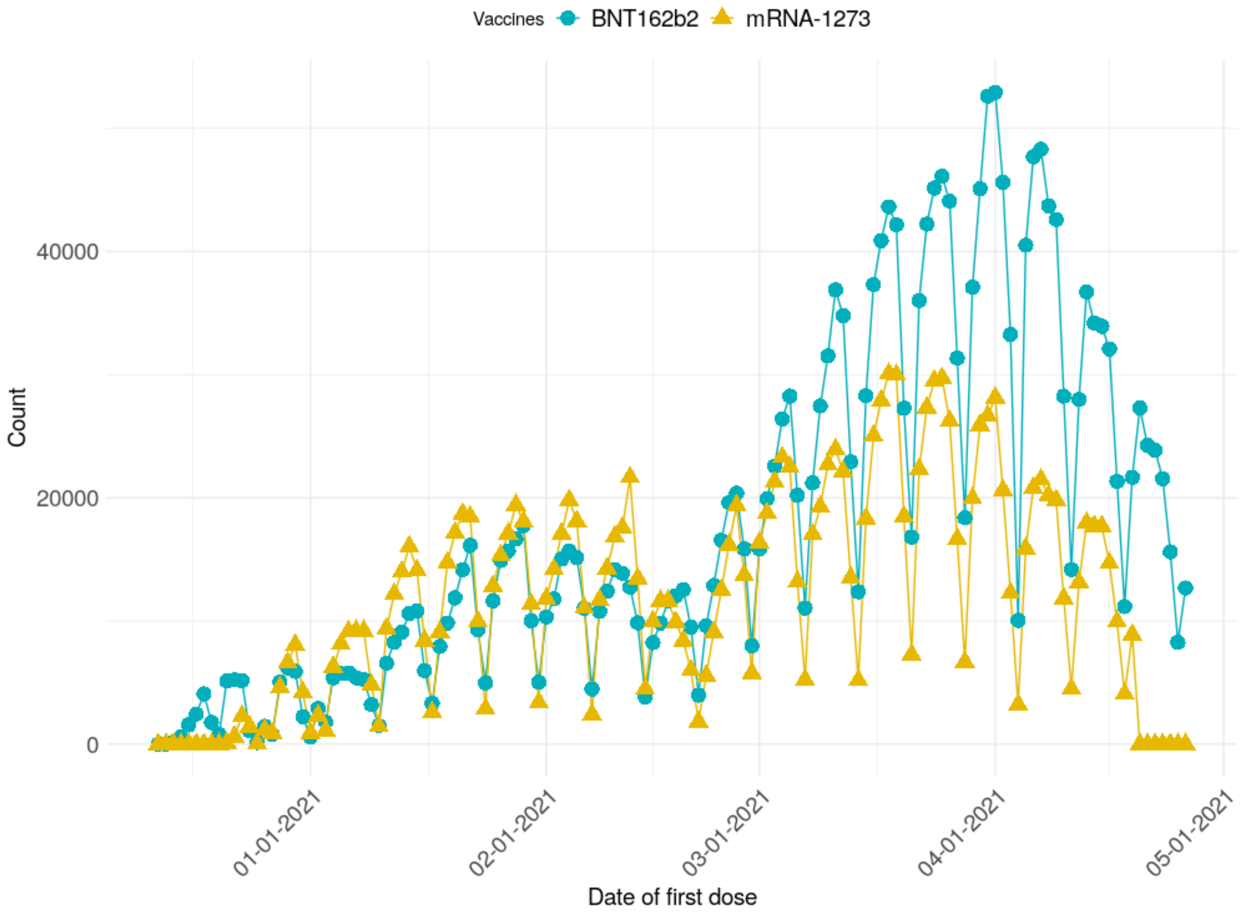

Figure S3. Sample population by state.
The sample (fully vaccinated) is nationally representative with variation between the two mRNA vaccines.

Sample population (count) by state with BNT162b2 vaccine

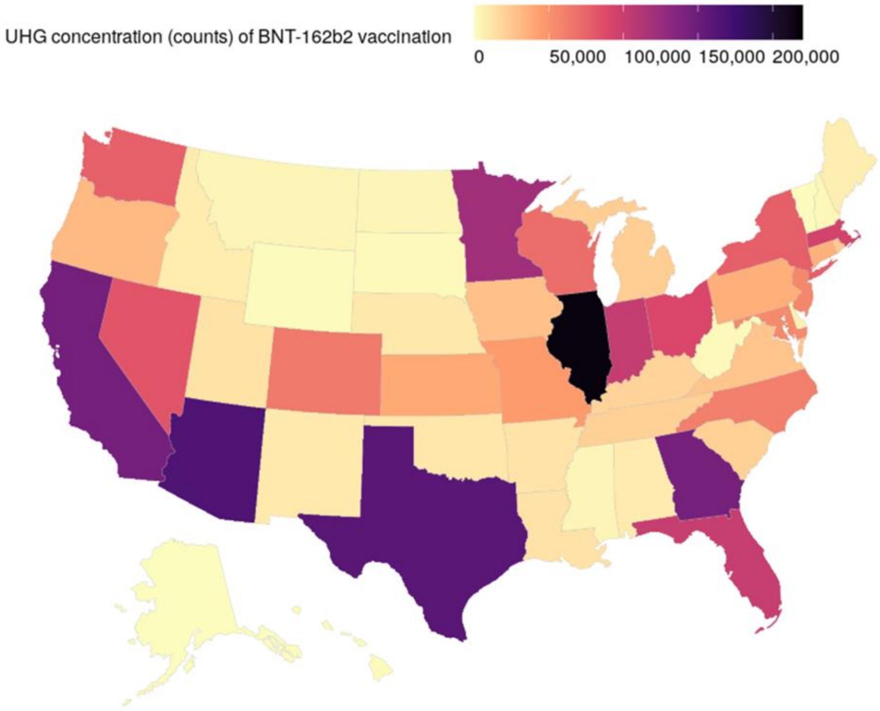

Sample population (count) by state with mRNA-173 vaccine

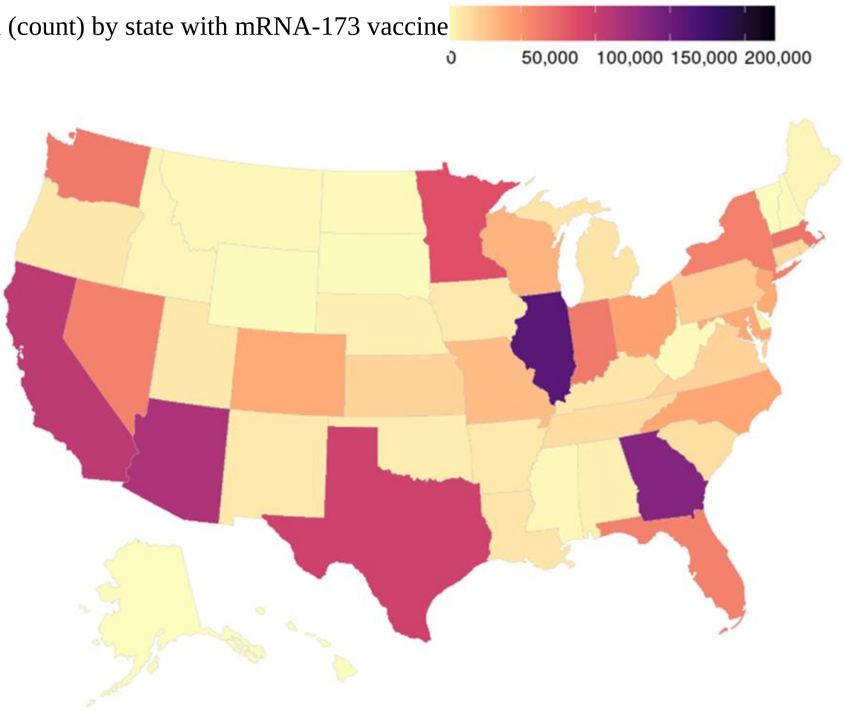

Figure S4. Incidence of events within 30 days of vaccination.
Adjusted odds ratio along with 95% confidence intervals, number needed to treat (NNT), and marginal rates (MR) of experiencing events per 1,000 people within 30d post vaccination (last dose + 14d) for BNT162b2 (denoted with a superscript of B) and mRNA-1273 (denoted with a superscript of m).

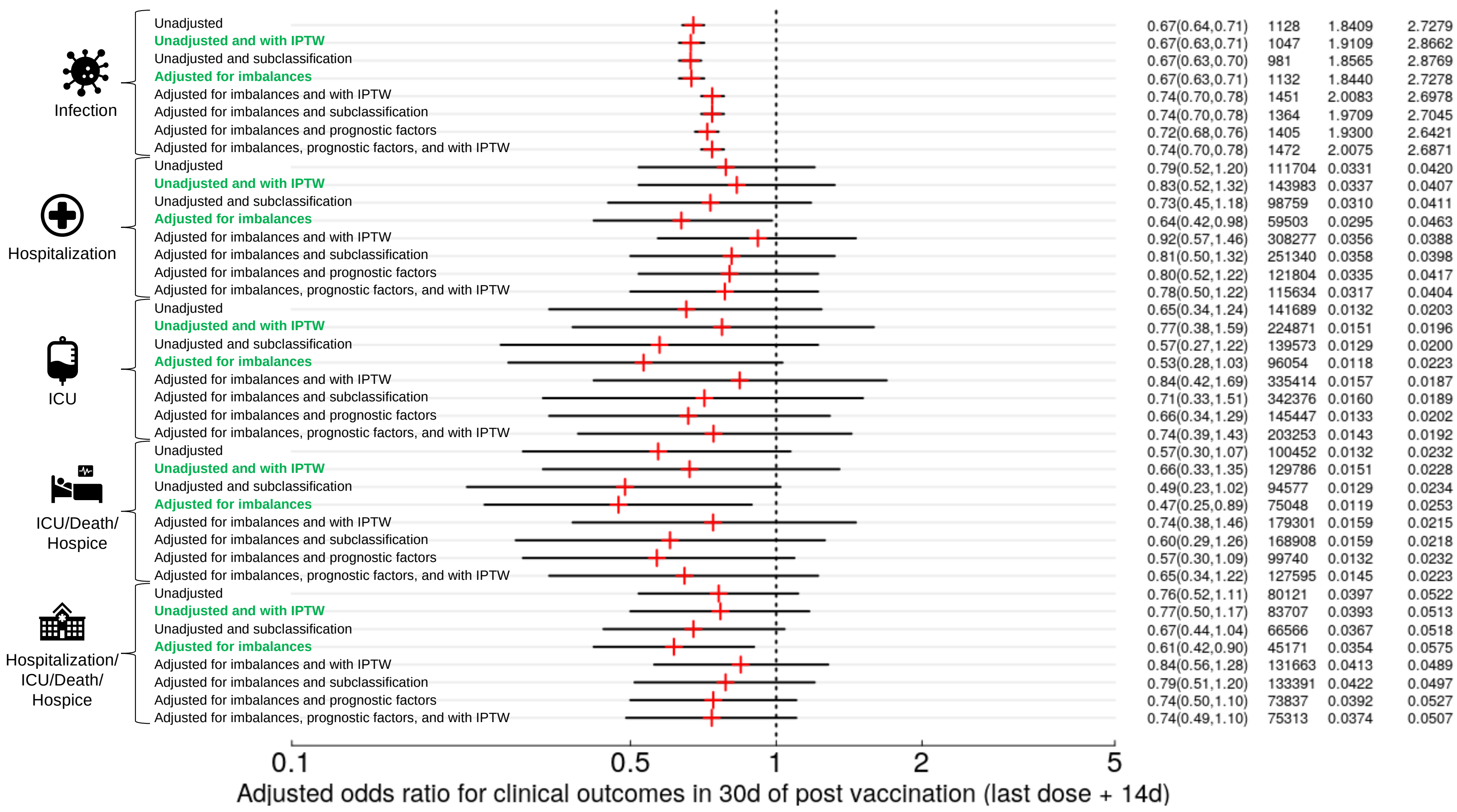

Outcome	Model	aOR (95% CI)	NNV	MR^m	MR^B
Infection	Unadjusted	0.67(0.64,0.71)	1128	1.8409	2.7279
	**Unadjusted and with IPTW**	0.67(0.63,0.71)	1047	1.9109	2.8662
	Unadjusted and subclassification	0.67(0.63,0.70)	981	1.8565	2.8769
	**Adjusted for imbalances**	0.67(0.63,0.71)	1132	1.8440	2.7278
	Adjusted for imbalances and with IPTW	0.74(0.70,0.78)	1451	2.0083	2.6978
	Adjusted for imbalances and subclassification	0.74(0.70,0.78)	1364	1.9709	2.7045
	Adjusted for imbalances and prognostic factors	0.72(0.68,0.76)	1405	1.9300	2.6421
	Adjusted for imbalances, prognostic factors, and with IPTW	0.74(0.70,0.78)	1472	2.0075	2.6871
Hospitalization	Unadjusted	0.79(0.52,1.20)	111704	0.0331	0.0420
	**Unadjusted and with IPTW**	0.83(0.52,1.32)	143983	0.0337	0.0407
	Unadjusted and subclassification	0.73(0.45,1.18)	98759	0.0310	0.0411
	**Adjusted for imbalances**	0.64(0.42,0.98)	59503	0.0295	0.0463
	Adjusted for imbalances and with IPTW	0.92(0.57,1.46)	308277	0.0356	0.0388
	Adjusted for imbalances and subclassification	0.81(0.50,1.32)	251340	0.0358	0.0398
	Adjusted for imbalances and prognostic factors	0.80(0.52,1.22)	121804	0.0335	0.0417
	Adjusted for imbalances, prognostic factors, and with IPTW	0.78(0.50,1.22)	115634	0.0317	0.0404
ICU	Unadjusted	0.65(0.34,1.24)	141689	0.0132	0.0203
	**Unadjusted and with IPTW**	0.77(0.38,1.59)	224871	0.0151	0.0196
	Unadjusted and subclassification	0.57(0.27,1.22)	139573	0.0129	0.0200
	**Adjusted for imbalances**	0.53(0.28,1.03)	96054	0.0118	0.0223
	Adjusted for imbalances and with IPTW	0.84(0.42,1.69)	335414	0.0157	0.0187
	Adjusted for imbalances and subclassification	0.71(0.33,1.51)	342376	0.0160	0.0189
	Adjusted for imbalances and prognostic factors	0.66(0.34,1.29)	145447	0.0133	0.0202
	Adjusted for imbalances, prognostic factors, and with IPTW	0.74(0.39,1.43)	203253	0.0143	0.0192
ICU/Death/ Hospice	Unadjusted	0.57(0.30,1.07)	100452	0.0132	0.0232
	**Unadjusted and with IPTW**	0.66(0.33,1.35)	129786	0.0151	0.0228
	Unadjusted and subclassification	0.49(0.23,1.02)	94577	0.0129	0.0234
	**Adjusted for imbalances**	0.47(0.25,0.89)	75048	0.0119	0.0253
	Adjusted for imbalances and with IPTW	0.74(0.38,1.46)	179301	0.0159	0.0215
	Adjusted for imbalances and subclassification	0.60(0.29,1.26)	168908	0.0159	0.0218
	Adjusted for imbalances and prognostic factors	0.57(0.30,1.09)	99740	0.0132	0.0232
	Adjusted for imbalances, prognostic factors, and with IPTW	0.65(0.34,1.22)	127595	0.0145	0.0223
Hospitalization/ ICU/Death/ Hospice	Unadjusted	0.76(0.52,1.11)	80121	0.0397	0.0522
	**Unadjusted and with IPTW**	0.77(0.50,1.17)	83707	0.0393	0.0513
	Unadjusted and subclassification	0.67(0.44,1.04)	66566	0.0367	0.0518
	**Adjusted for imbalances**	0.61(0.42,0.90)	45171	0.0354	0.0575
	Adjusted for imbalances and with IPTW	0.84(0.56,1.28)	131663	0.0413	0.0489
	Adjusted for imbalances and subclassification	0.79(0.51,1.20)	133391	0.0422	0.0497
	Adjusted for imbalances and prognostic factors	0.74(0.50,1.10)	73837	0.0392	0.0527
	Adjusted for imbalances, prognostic factors, and with IPTW	0.74(0.49,1.10)	75313	0.0374	0.0507

Figure S5. Incidence of events within 60 days of vaccination.
Adjusted odds ratio along with 95% confidence intervals, number needed to treat (NNT), and marginal rates (MR) of experiencing events within 60d post vaccination (last dose + 14d) for BNT162b2 (denoted with a superscript of B) and mRNA-1273 (denoted with a superscript of m).

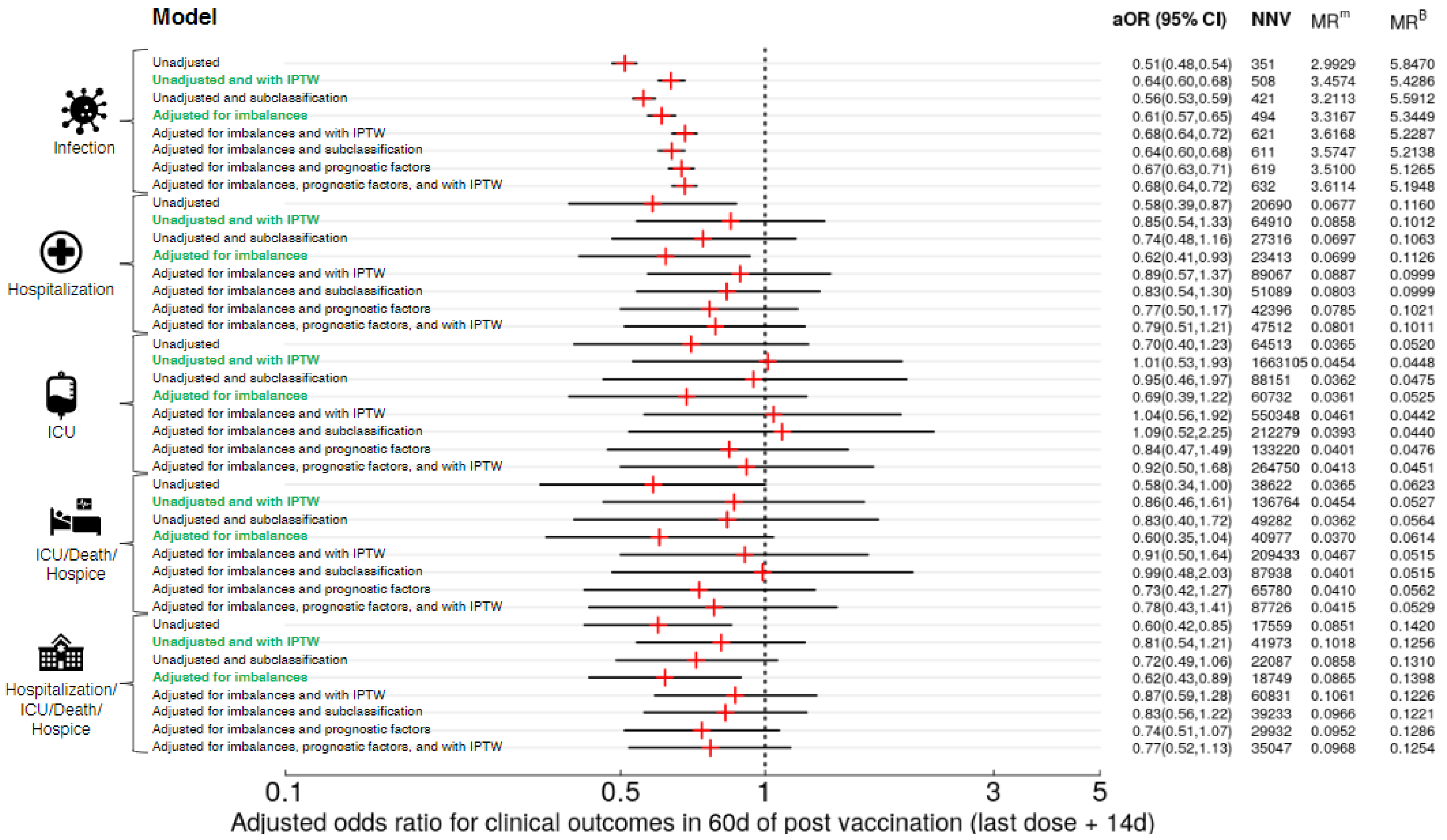

Figure S6. Incidence of events within 90 days of vaccination.
Adjusted odds ratio along with 95% confidence intervals, number needed to treat (NNT), and marginal rates (MR) of experiencing events within 90d post vaccination (last dose + 14d) for BNT162b2 (denoted with a superscript of B) and mRNA-1273 (denoted with a superscript of m).

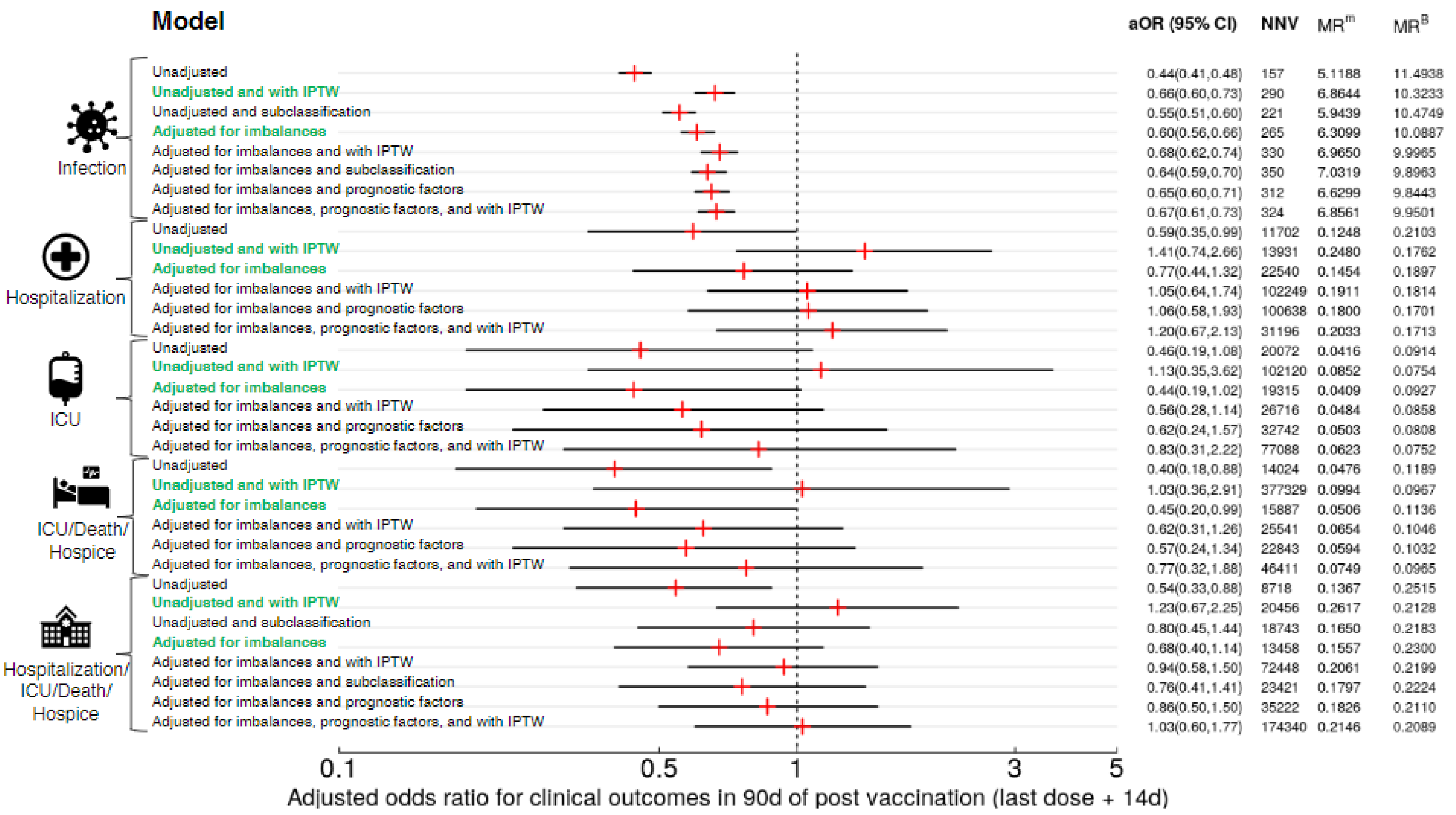

Figure S7. Hazard ratios for comorbidities.

Heatmap showing adjusted hazard ratios for comorbidities considered in modeling. Blank boxes without numbers indicate the variable was either not selected in the variable screening step or not found to be statistically significant in the multivariable model.

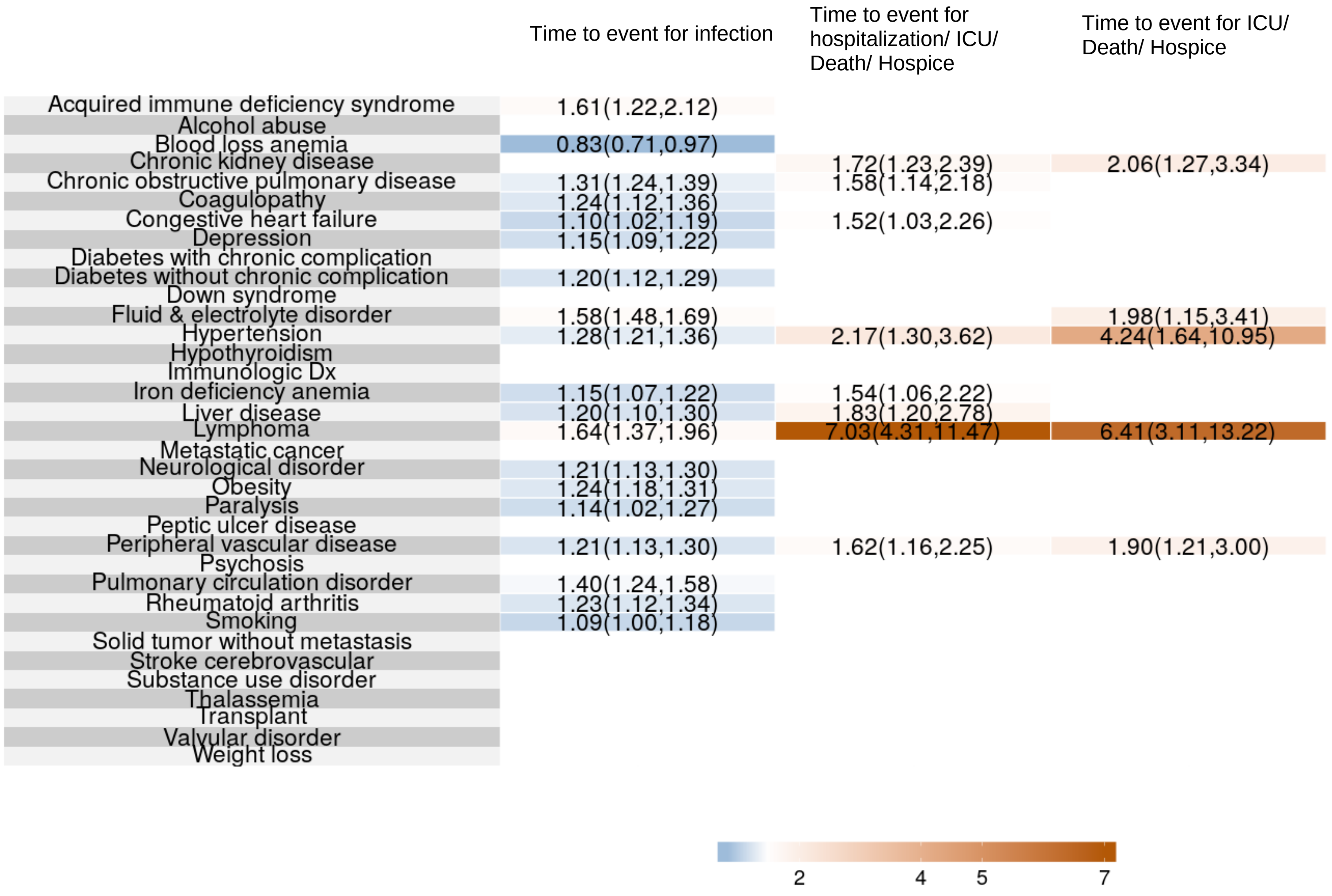

Figure S8. Predicted survival probabilities.
Predicted survival probabilities of a typical (prediction at mode) patient based on the adjusted model for imbalances along with IPTW with respect to (A) SARS-CoV-2 infection and (B) hospitalization/ICU/death/hospice whichever occurring first.

Time-to-infection	Time-to-hospitalization, ICU, death, or discharge to hospice

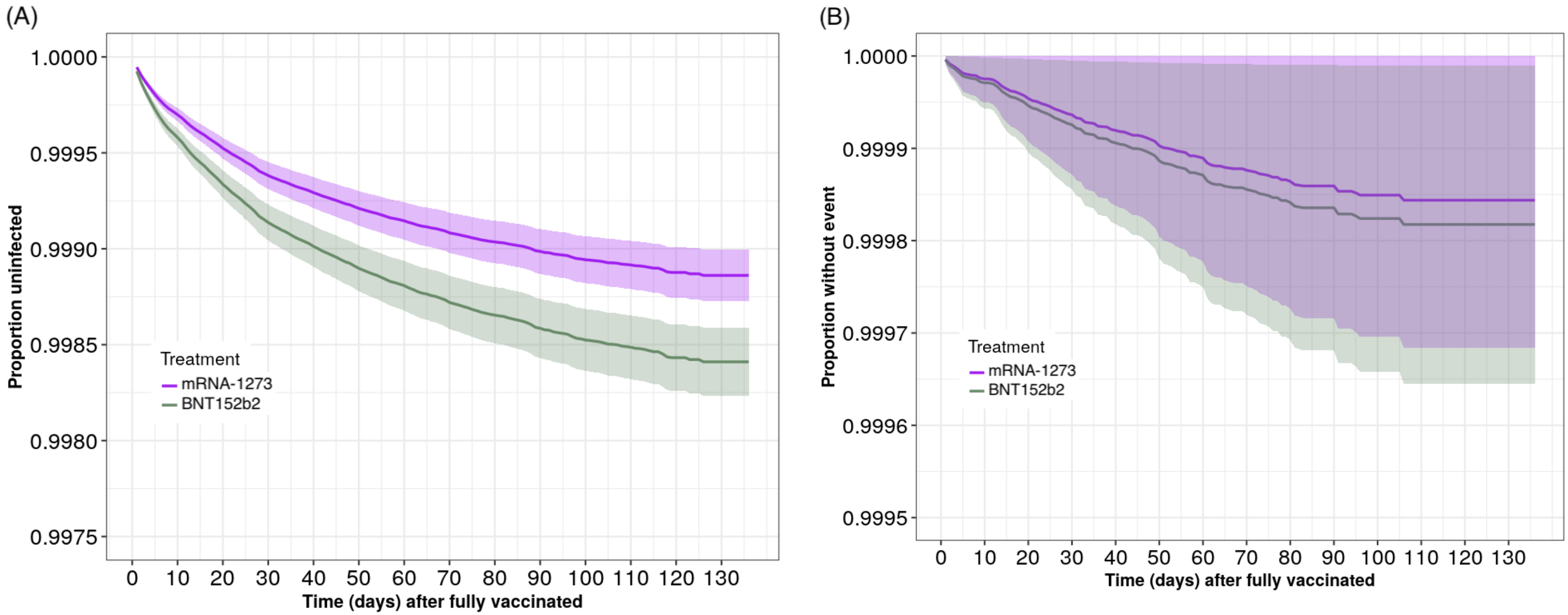

Table S2. Differences of covariates.

Standardized mean differences of covariates in unweighted and weighted (IPTW) samples between BNT162b2 and mRNA-1273 vaccinated individuals.

	30 DAY POST-VACCINATION BINARY OUTCOME		60 DAY POST-VACCINATION BINARY OUTCOME		90 DAY POST-VACCINATION BINARY OUTCOME		POST-VACCINATION TIME-TO-EVENT	
	Unweighted	IPTW	Unweighted	IPTW	Unweighted	IPTW	Unweighted	IPTW
Age†¶‡	0.21	<0.001	0.083	0.002	0.054	0.01	0.267	<0.001
Insurance Type†¶‡	0.259	0.001	0.135	0.002	0.006	0.011	0.279	<0.001
Acquired immune deficiency syndrome†‡	0.008	<0.001	0.006	<0.001	0.005	0.004	0.001	<0.001
Alcohol abuse†‡	0.001	<0.001	0.007	0.001	0.029	0.008	0.006	<0.001
Iron deficiency anemia†‡	0.045	0.001	0.002	0.002	0.048	0.011	0.058	<0.001
Rheumatoid arthritis†‡	0.027	<0.001	0.015	0.001	0.006	0.001	0.039	<0.001
Blood loss anemia†‡	0.005	<0.001	0.005	0.001	0.015	0.001	0.007	<0.001
Congestive heart failure†‡	0.044	0.001	0.003	0.002	0.078	0.012	0.06	<0.001
Chronic obstructive pulmonary disease†‡	0.05	<0.001	0.017	0.001	0.025	0.007	0.068	<0.001
Coagulopathy†‡	0.018	<0.001	0.001	<0.001	0.022	0.003	0.025	<0.001
Depression†‡	0.003	0.001	0.025	0.001	0.075	0.016	0.015	0.001
Diabetes without chronic complication†‡	0.076	0.001	0.04	0.002	0.005	0.01	0.102	<0.001
Diabetes with chronic complication†‡	0.072	0.001	0.031	0.001	0.016	0.01	0.096	<0.001
Substance use disorder†	0.005	0.001	0.017	0.002	0.06	0.019	0.014	0.001
Hypertension†‡	0.13	0.001	0.063	0.002	0.02	0.011	0.171	<0.001
Hypothyroidism†‡	0.046	<0.001	0.015	0.001	0.017	0.005	0.063	<0.001
Liver disease†‡	0.011	<0.001	0.005	<0.001	0.002	0.003	0.023	<0.001
Lymphoma†‡	0.006	<0.001	0.003	<0.001	0.004	<0.001	0.01	<0.001
Fluid & electrolyte disorder†‡	0.032	0.001	0.009	0.002	0.066	0.013	0.047	<0.001
Metastatic cancer†‡	0.002	<0.001	0.005	<0.001	0.007	0.001	0.01	<0.001
Neurological disorder†‡	0.019	0.001	0.036	0.002	0.122	0.024	0.038	<0.001
Obesity†‡	0.038	<0.001	0.037	0.001	0.033	0.003	0.058	<0.001
Paralysis†‡	0.002	0.002	0.029	0.004	0.077	0.032	0.014	0.001
Peripheral vascular disease†‡	0.063	0.001	0.005	0.002	0.096	0.021	0.082	<0.001
Psychosis†‡	0.005	0.001	0.025	0.001	0.068	0.017	0.007	<0.001
Pulmonary circulation disorder†‡	0.008	0.001	0.007	0.001	0.022	0.006	0.015	<0.001
Chronic kidney disease†‡	0.074	0.001	0.016	0.002	0.053	0.012	0.091	<0.001
Solid tumor without metastasis†‡	0.027	<0.001	0.003	<0.001	0.01	0.001	0.043	<0.001
Peptic ulcer disease†‡	0.01	<0.001	0.004	<0.001	0.007	0.004	0.014	<0.001
Valvular disorder†‡	0.052	0.001	0.016	0.001	0.006	0.003	0.064	<0.001
Weight loss†‡	0.005	0.001	0.03	0.002	0.087	0.02	0.014	0.001
Stroke cerebrovascular†	0.053	0.001	<0.001	0.002	0.056	0.014	0.07	<0.001
Down syndrome†	0.001	<0.001	<0.001	<0.001	0.005	<0.001	0.002	<0.001
Thalassemia†	0.001	<0.001	<0.001	<0.001	<0.001	<0.001	0.001	<0.001
Smoking†	0.031	<0.001	0.02	<0.001	0.004	0.002	0.042	<0.001
Transplant†	0.001	<0.001	<0.001	<0.001	0.004	<0.001	0.003	<0.001
Elixhauser mortality score	0.053	0.003	0.015	0.006	0.104	0.024	0.076	0.002

Elixhauser readmission score	0.076	0.001	0.01	0.003	0.123	0.027	0.11	<0.001
Nursing facility/SNF†‡	0.03	0.006	0.057	0.01	0.082	0.039	0.019	0.004
Immunologic Rx	0.08	0.011	0.046	0.007	0.001	0.017	0.092	0.011
Immunologic Dx†‡	0.04	<0.001	0.021	0.001	0.022	0.002	0.064	<0.001
Gender†‡	0.02	0.001	0.03	<0.001	0.058	0.001	0.025	<0.001
Residence by state†¶‡	0.223	0.011	0.265	0.011	0.494	0.028	0.194	0.013
Residence by region*	0.07	0.001	0.132	0.003	0.318	0.011	0.058	0.002
First dose administered before February 1, 2021†¶*	0.244	<0.001	0.149	0.001	0.186	0.009	0.237	<0.001
SES index†¶‡	0.171	0.001	0.148	0.002	0.031	0.005	0.206	0.001
Prior any Covid Dx (ER/IP/ICU/Dx) †¶	0.03	0.002	0.064	0.003	0.105	0.02	0.016	0.001
Prior positive PCR	0.01	0.012	0.011	0.023	0.012	0.041	0.007	0.008
Urban/ Rural residence†¶‡	0.286	0.001	0.264	0.001	0.164	0.005	0.282	<0.001

†Adjusted in propensity score model. Variables are adjusted in outcome regression models (as in equation SM 7-8).
¶‡Variables are adjusted in outcome regression models (as in equation SM 4-6).
**Residence by region is used instead of residence by state in Cox-PH model.*
***For the 90-day outcome the date was adjusted to "First dose administered before January 10, 2021."*
‡Variables are adjusted in negative outcome regression models (as in equation SM 7-8).

Table S3. Strength of confounders and selection bias.

(Top panel) Summary measures determining the overall strength of unmeasured confounder (E-value) and selection bias required to explain away the treatment effects (i.e., aOR of mRNA-1273 vs BNT-162b2) with respect to SARS-CoV-2 infection within 30-day, 60-day, and 90-day interval under different models (except subclassification models SM3 and SM6). Corresponding summary measures of the upper bounds of 95% CIs of aORs are reported in parenthesis. (Bottom panel) displays the empirical null distribution of bias estimated from the coefficient estimates of treatment effect using seven negative control outcomes.

		30d		60d		90d			Time to event	
		E-value (CI)	Selection (CI)	E-value (CI)	Selection (CI)	E-value (CI)	Selection (CI)		E-value (CI)	Selection (CI)
Bounding values for unmeasured confounder and selection bias	(SM1)	2.33(2.16)	1.73(1.65)	3.33(3.10)	2.15(2.06)	3.95(3.60)	2.37(2.25)		2.08(1.95)	1.62(1.55)
	(SM2)	2.37(2.19)	1.75(1.67)	2.52(2.32)	1.82(1.73)	2.39(2.10)	1.76(1.63)		2.38(2.24)	1.76(1.69)
	(SM3)	2.37(2.19)	1.75(1.67)	2.98(2.76)	2.01(1.92)	3.01(2.71)	2.02(1.90)		-	-
	(SM4)	2.36(2.18)	1.75(1.66)	2.67(2.47)	1.88(1.79)	2.69(2.42)	1.89(1.77)		2.27(2.13)	1.71(1.64)
	(SM5)	2.05(1.88)	1.60(1.52)	2.30(2.11)	1.72(1.63)	2.31(2.05)	1.72(1.60)		2.14(2.00)	1.64(1.58)
	(SM6)	2.05(1.88)	1.60(1.52)	2.51(2.31)	1.81(1.72)	2.51(2.23)	1.81(1.69)		-	-
	(SM7)	2.12(1.95)	1.64(1.55)	2.35(2.16)	1.74(1.65)	2.44(2.18)	1.78(1.66)		2.01(1.88)	1.58(1.52)
	(SM8)	2.05(1.88)	1.60(1.52)	2.30(2.11)	1.72(1.63)	2.37(2.10)	1.75(1.63)		2.05(1.91)	1.60(1.53)
Negative controls		-0.0202 (0.0360)		-0.0251 (0.0507)		-0.0235 (0.0735)			-0.0082(0.0291)	

Table S4. Baseline characteristics among those aged 65 and older. Descriptive statistics for baseline characteristics (demographics, socio-economic, comorbidities, negative controls, study outcomes, and other prognostic factors) among individuals aged at least 65 and fully vaccinated by BNT162b2 or mRNA-1273 by June 1, 2021.

	POST-VACCINATION TIME-TO-EVENT	
	BNT162B26	MRNA-12737
N	529138	554929
Age, mean (SD)	74.27 (7.36)	74.55 (7.09)
65–69, N (%)	169,161 (32.0)	159,330 (28.7)
70–74, N (%)	141,787 (26.8)	154,884 (27.9)
75–79, N (%)	99,802 (18.9)	113,859 (20.5)
80–84, N (%)	60,991 (11.5)	68,607 (12.4)
85+, N (%)	57,397 (10.8)	58,249 (10.5)
Insurance type, N (%)		
Medicare Advantage	379,068 (71.6)	432,257 (77.9)
Commercial	150,070 (28.4)	122,672 (22.1)
Acquired immune deficiency syndrome (%)	899 (0.2)	875 (0.2)
Alcohol use disorder (%)	6,866 (1.3)	7,058 (1.3)
Iron deficiency anemia (%)	68,831 (13.0)	73,192 (13.2)
Rheumatoid arthritis (%)	29,982 (5.7)	32,502 (5.9)
Blood loss anemia (%)	6,128 (1.2)	6,250 (1.1)
Congestive heart failure (%)	42,477 (8.0)	44,100 (7.9)
Chronic obstructive pulmonary disease (%)	79,865 (15.1)	88,106 (15.9)
Coagulopathy (%)	15,113 (2.9)	15,539 (2.8)
Depression (%)	66,006 (12.5)	64,771 (11.7)
Diabetes without chronic complication (%)	113,510 (21.5)	126,989 (22.9)
Diabetes with chronic complication (%)	92,515 (17.5)	103,286 (18.6)
Substance use disorder (%)	8,712 (1.6)	8,252 (1.5)
Hypertension (%)	332,706 (62.9)	361,039 (65.1)
Hypothyroidism (%)	95,591 (18.1)	102,617 (18.5)
Liver disease (%)	24,312 (4.6)	25,621 (4.6)
Lymphoma (%)	5,105 (1.0)	4,983 (0.9)
Fluid & electrolyte disorder (%)	48,042 (9.1)	48,705 (8.8)
Metastatic cancer (%)	7,465 (1.4)	6,895 (1.2)
Neurological disorder (%)	52,105 (9.8)	49,277 (8.9)
Obesity (%)	88,719 (16.8)	100,668 (18.1)
Paralysis (%)	8,047 (1.5)	7,073 (1.3)
Peripheral vascular disease (%)	77,295 (14.6)	80,542 (14.5)
Psychosis (%)	20,667 (3.9)	19,077 (3.4)
Pulmonary circulation disorder (%)	6,981 (1.3)	6,726 (1.2)
Chronic kidney disease (%)	71,802 (13.6)	78,740 (14.2)
Solid tumor without metastasis (%)	53,568 (10.1)	54,382 (9.8)
Peptic ulcer disease (%)	4,399 (0.8)	4,645 (0.8)
Valvular disorder (%)	52,930 (10.0)	57,344 (10.3)
Weight loss (%)	19,721 (3.7)	17,793 (3.2)
Stroke cerebrovascular (%)	64,561 (12.2)	67,451 (12.2)
Down syndrome (%)	7 (0.0)	7 (0.0)
Thalassemia (%)	672 (0.1)	654 (0.1)
Smoking (%)	35,996 (6.8)	39,881 (7.2)
Transplant (%)	633 (0.1)	622 (0.1)
Elixhauser mortality score, mean (SD)	3.35 (6.71)	3.24 (6.42)

Elixhauser readmission score, mean (SD)	11.11 (15.65)	10.99 (15.00)
Transferred from nursing facility/SNF (%)	2,201 (0.4)	717 (0.1)
Immunologic Rx (%)	190,012 (35.9)	211,546 (38.1)
Immunologic Dx (%)	207,253 (39.2)	218,695 (39.4)
Sex		
Female (%)	302,497 (57.2)	315,047 (56.8)
Male (%)	226,641 (42.8)	239,882 (43.2)
Residence by region (%)		
Midwest	175,119 (33.1)	149,772 (27.0)
Northeast	61,835 (11.7)	60,972 (11.0)
South	139,891 (26.4)	177,871 (32.1)
West	152,293 (28.8)	166,314 (30.0)
Residence class (%)		
Rural	94,412 (17.8)	180,541 (32.5)
Urban	210,091 (39.7)	166,773 (30.1)
Semi-urban	224,635 (42.5)	207,615 (37.4)
First dose administered before Feb 1, 2021 (%)	140,771 (26.6)	186,088 (33.5)
SES index, mean (SD)	53.09 (2.81)	52.34 (3.13)
Prior any Covid Dx (ER/IP/ICU) (%)	36,593 (6.9)	32,731 (5.9)
Prior positive PCR (%)	6,403 (1.2)	6,333 (1.1)
Negative outcomes, N (%)		
Bilateral prim osteoarthritis knee	14,426 (2.7)	14,758 (2.7)
Dry eye syndrome	0 (0.0)	0 (0.0)
Frequency of micturition	19,307 (3.6)	19,621 (3.5)
Presbyopia	27,536 (5.2)	30,052 (5.4)
Seborrheic keratosis	0 (0.0)	0 (0.0)
Sensorineural hear loss bilateral	19,340 (3.7)	19,500 (3.5)
Tinea unguium	36,370 (6.9)	33,492 (6.0)
UTI site not specified	38,978 (7.4)	41,322 (7.4)
Agerel nuclear cataract bilateral	53,060 (10.0)	56,846 (10.2)

Figure S9. Incidence of events 10d, 30d, 50d, 70d, and 90d post-vaccination for those aged 65 and younger. Adjusted hazard ratios (aHRs) along with 95% confidence intervals, number needed to treat (NNT), and predicted marginal rates per 1,000 individuals (in parenthesis) of experiencing events at 10d, 30d, 50d, 70d, and 90d post-vaccination (last dose + 14d) for mRNA-1273 (Moderna) and BNT162b2 (Pfizer), respectively (separated by “/”). Time-to-event for adverse outcomes (top-panel) ICU/death/hospice and (bottom-panel) hospitalization/ICU/death/hospice whichever occurring first among vaccinated individuals aged ≥65.

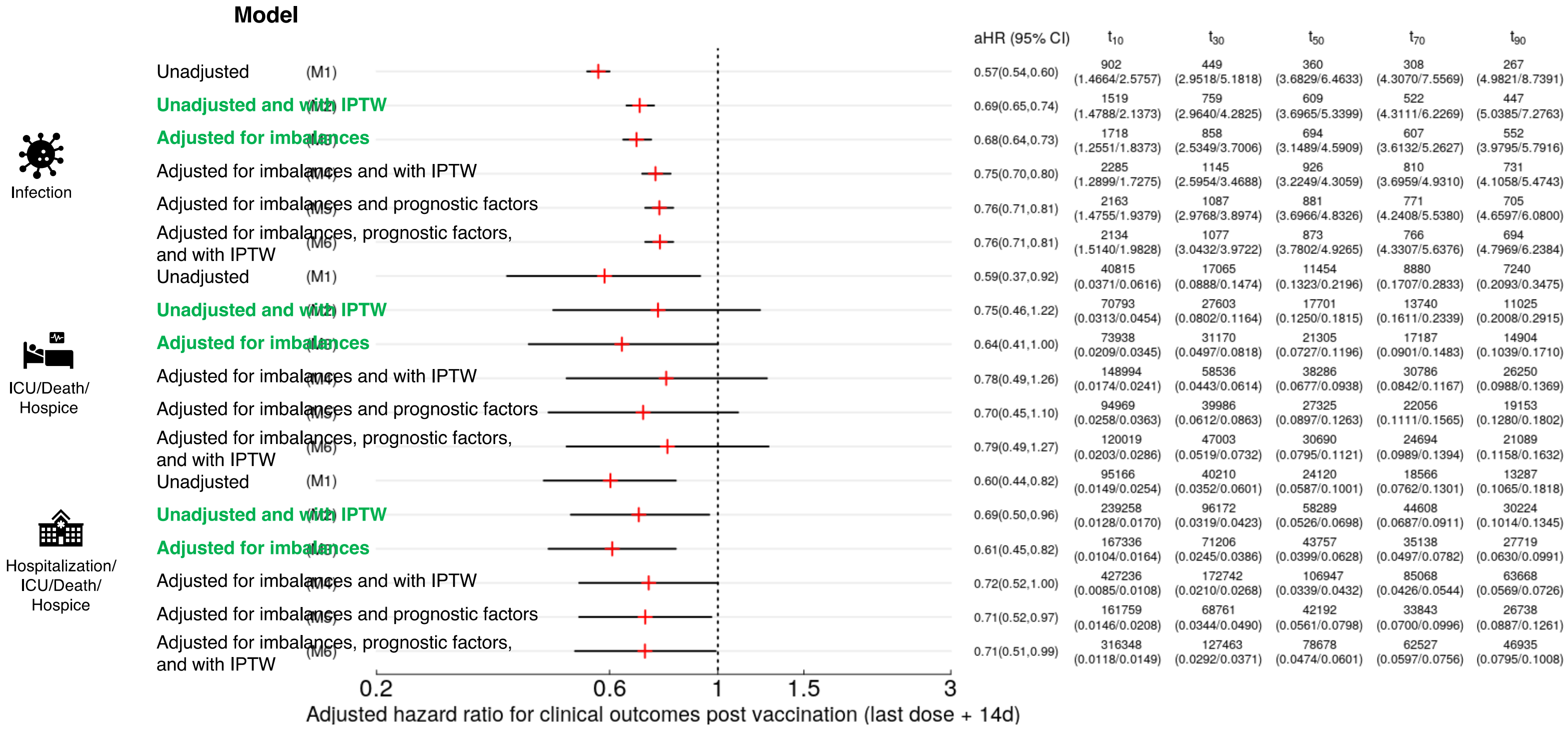

Table S5. Baseline characteristics among those aged 65 and younger. Descriptive statistics for baseline characteristics (demographics, socio-economic, comorbidities, negative controls, study outcomes, and other prognostic factors) among individuals aged 64 and younger and fully vaccinated by BNT162b2 or mRNA-1273 by June 1, 2021.

	POST-VACCINATION TIME-TO-EVENT	
	BNT162B26	**MRNA-12737**
N	1,835,467	1,043,521
Age, mean (SD)	43.86 (13.04)	45.20 (12.71)
18-24, N (%)	168,893 (9.2)	71,792 (6.9)
25-29, N (%)	147,969 (8.1)	79,056 (7.6)
30-34, N (%)	183,193 (10.0)	98,126 (9.4)
35-39, N (%)	200,901 (10.9)	111,811 (10.7)
40-44, N (%)	205,561 (11.2)	116,946 (11.2)
45-49, N (%)	204,181 (11.1)	115,928 (11.1)
50-54, N (%)	229,689 (12.5)	133,745 (12.8)
55-59, N (%)	243,091 (13.2)	149,095 (14.3)
60-64, N (%)	251,989 (13.7)	167,022 (16.0)
Insurance type, N (%)		
Medicare Advantage	26,955 (1.5)	28,046 (2.7)
Commercial	1,808,512 (98.5)	1,015,475 (97.3)
Acquired immune deficiency syndrome (%)	5,080 (0.3)	3,214 (0.3)
Alcohol use disorder (%)	16,201 (0.9)	9,447 (0.9)
Iron deficiency anemia (%)	65,618 (3.6)	40,362 (3.9)
Rheumatoid arthritis (%)	38,737 (2.1)	25,148 (2.4)
Blood loss anemia (%)	10,530 (0.6)	5,918 (0.6)
Congestive heart failure (%)	11,982 (0.7)	8,666 (0.8)
Chronic obstructive pulmonary disease (%)	102,063 (5.6)	65,370 (6.3)
Coagulopathy (%)	12,031 (0.7)	7,240 (0.7)
Depression (%)	154,362 (8.4)	91,253 (8.7)
Diabetes without chronic complication (%)	106,491 (5.8)	72,274 (6.9)
Diabetes with chronic complication (%)	72,567 (4.0)	50,534 (4.8)
Substance use disorder (%)	12,436 (0.7)	8,282 (0.8)
Hypertension (%)	317,680 (17.3)	204,851 (19.6)
Hypothyroidism (%)	120,648 (6.6)	73,868 (7.1)
Liver disease (%)	44,381 (2.4)	27,212 (2.6)
Lymphoma (%)	4,093 (0.2)	2,311 (0.2)
Fluid & electrolyte disorder (%)	35,792 (2.0)	22,847 (2.2)
Metastatic cancer (%)	6,689 (0.4)	3,894 (0.4)
Neurological disorder (%)	38,484 (2.1)	24,203 (2.3)
Obesity (%)	196,581 (10.7)	123,495 (11.8)
Paralysis (%)	4,781 (0.3)	3,268 (0.3)
Peripheral vascular disease (%)	19,576 (1.1)	13,471 (1.3)
Psychosis (%)	44,300 (2.4)	26,738 (2.6)
Pulmonary circulation disorder (%)	4,996 (0.3)	3,154 (0.3)
Chronic kidney disease (%)	18,174 (1.0)	12,913 (1.2)
Solid tumor without metastasis (%)	41,096 (2.2)	23,780 (2.3)
Peptic ulcer disease (%)	4,141 (0.2)	2,557 (0.2)
Valvular disorder (%)	27,917 (1.5)	17,525 (1.7)
Weight loss (%)	14,381 (0.8)	7,976 (0.8)
Stroke cerebrovascular (%)	24,780 (1.4)	16,143 (1.5)
Down syndrome (%)	412 (0.0)	322 (0.0)

Thalassemia (%)	1,838 (0.1)	1,005 (0.1)
Smoking (%)	60,874 (3.3)	39,703 (3.8)
Transplant (%)	853 (0.0)	493 (0.0)
Elixhauser mortality score, mean (SD)	0.62 (2.60)	0.67 (2.72)
Elixhauser readmission score, mean (SD)	2.78 (6.82)	3.08 (7.39)
Transferred from nursing facility/SNF (%)	264 (0.0)	115 (0.0)
Immunologic Rx (%)	306,783 (16.7)	185,874 (17.8)
Immunologic Dx (%)	349,387 (19.0)	201,829 (19.3)
Sex		
Female (%)	961,578 (52.4)	559,360 (53.6)
Male (%)	873,889 (47.6)	484,161 (46.4)
Residence by region (%)		
Midwest	577,448 (31.5)	317,930 (30.5)
Northeast	274,042 (14.9)	167,455 (16.0)
South	532,785 (29.0)	303,367 (29.1)
West	451,192 (24.6)	254,769 (24.4)
Residence class (%)		
Rural	221,577 (12.1)	204,391 (19.6)
Urban	823,446 (44.9)	410,130 (39.3)
Semi-urban	790,444 (43.1)	429,000 (41.1)
First dose administered before Feb 1, 2021 (%)	177,092 (9.6)	173,284 (16.6)
SES index, mean (SD)	53.87 (2.91)	53.47 (3.04)
Prior any Covid Dx (ER/IP/ICU) (%)	132,484 (7.2)	75,160 (7.2)
Prior positive PCR (%)	33,652 (1.8)	19,262 (1.8)
Negative outcomes, N (%)		
Bilateral prim osteoarthritis knee	10,356 (0.6)	6,868 (0.7)
Dry eye syndrome	3 (0.0)	2 (0.0)
Frequency of micturition	25,460 (1.4)	15,103 (1.4)
Presbyopia	21,227 (1.2)	132,272 (1.3)
Seborrheic keratosis	0 (0.0)	0 (0.0)
Sensorineural hear loss bilateral	11,554 (0.6)	6,804 (0.7)
Tinea unguium	17,961 (1.0)	11,181 (1.1)
UTI site not specified	45,879 (2.5)	28,919 (2.8)
Agerel nuclear cataract bilateral	24,990 (1.4)	15,866 (1.5)

Figure S10. Incidence of events 10d, 30d, 50d, 70d, and 90d post-vaccination for those aged 65 and older. Adjusted hazard ratios (aHRs) along with 95% confidence intervals, number needed to treat (NNT), and predicted marginal rates per 1,000 individuals (in parenthesis) of experiencing events at 10d, 30d, 50d, 70d, and 90d post vaccination (last dose + 14d) for mRNA-1273 (Moderna) and BNT162b2 (Pfizer), respectively (separated by “/”). Time-to-event for adverse outcomes (top-panel) ICU/death/hospice and (bottom-panel) hospitalization/ICU/death/hospice whichever occurring first among vaccinated individuals aged less than 65.

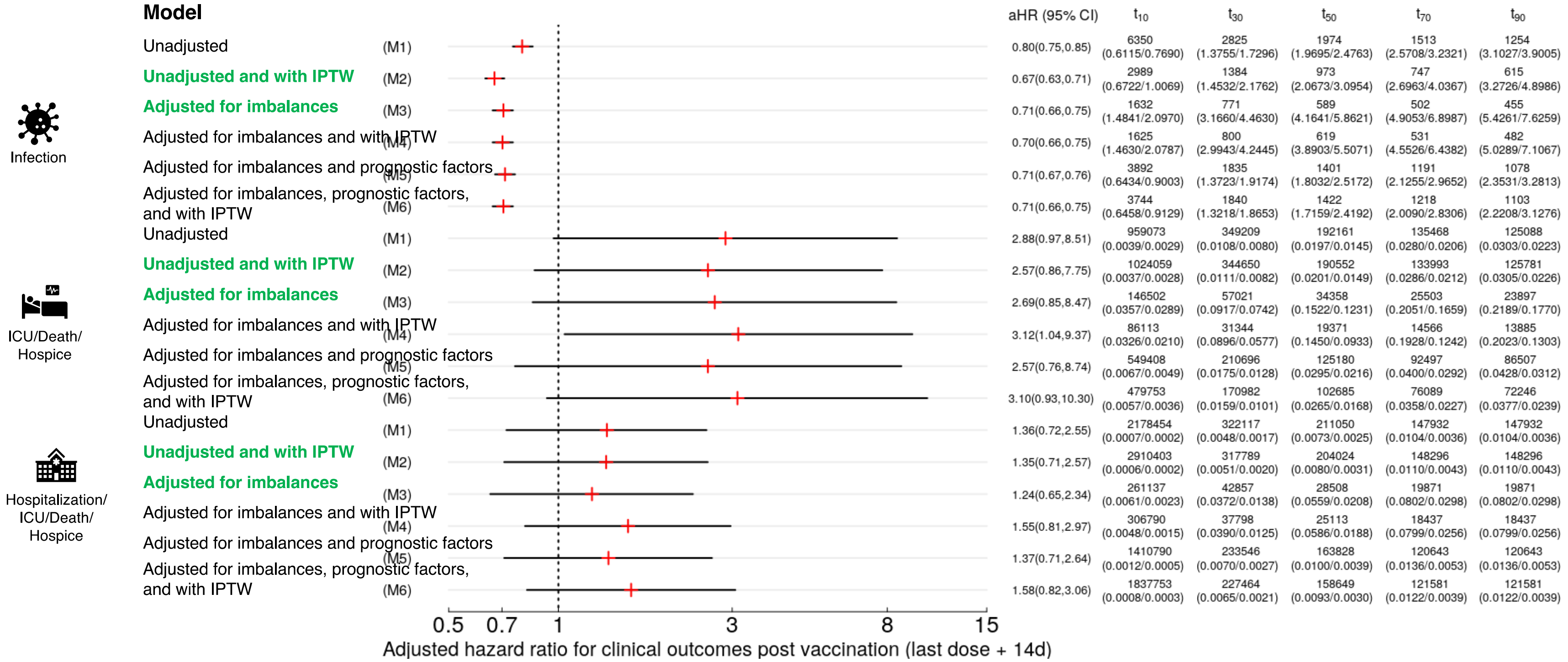

Figure S11. Incidence of events 10d, 30d, 50d, 70d, and 90d post-vaccination among vaccinated individuals without prior Covid-19 diagnosis.

Adjusted hazard ratios (aHRs) along with 95% confidence intervals, number needed to treat (NNT), and predicted marginal rates per 1,000 individuals (in parenthesis) of experiencing events at 10d, 30d, 50d, 70d, and 90d post vaccination (last dose + 14d) for mRNA-1273 (Moderna) and BNT162b2 (Pfizer), respectively (separated by “/”). Time-to-event for adverse outcomes (top-panel) ICU/death/hospice and (bottom-panel) hospitalization/ICU/death/hospice whichever occurring first among vaccinated individuals without prior diagnosis of Covid.

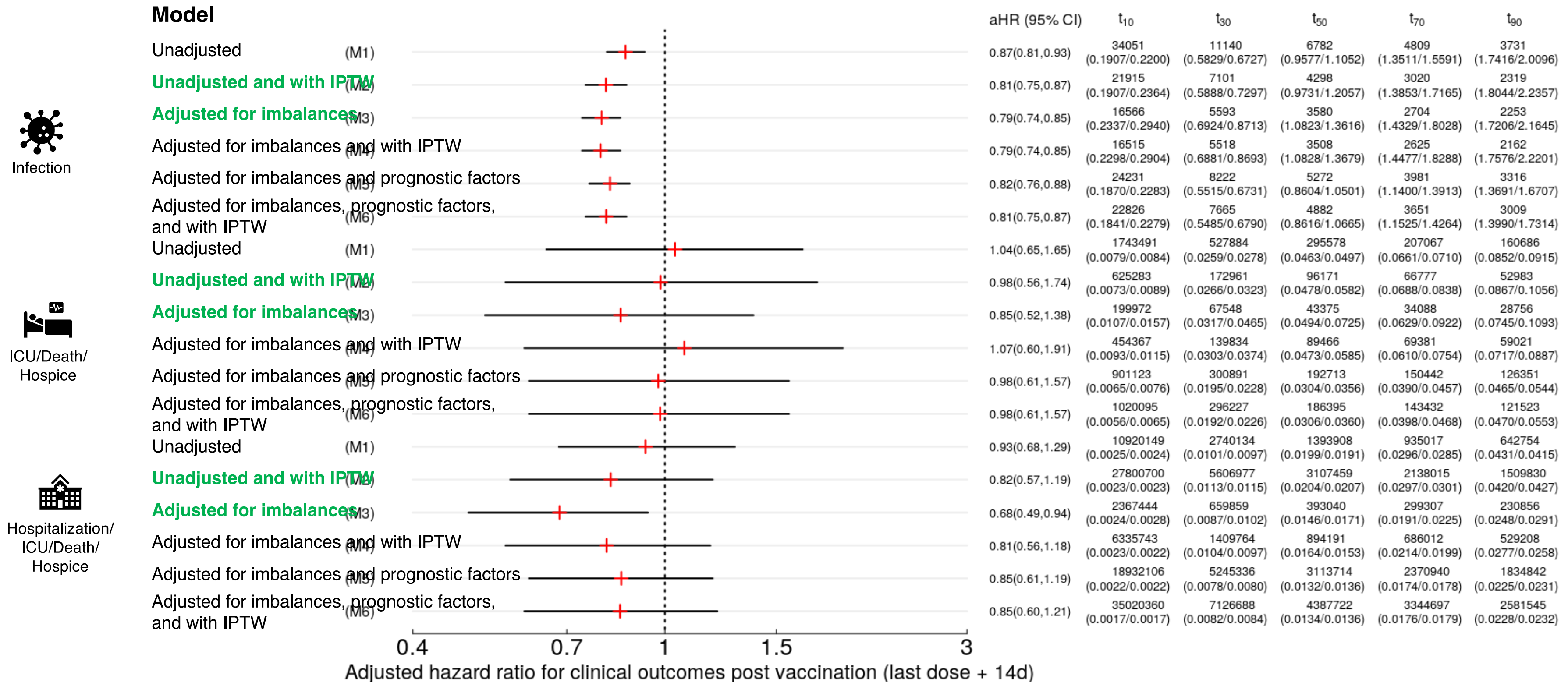

Supplemental Material

Results for population of vaccinated people who became infected

Table S6. Baseline characteristics among fully vaccinated individuals who became infected with Covid-19.

Descriptive statistics for baseline characteristics (demographics, socio-economic, comorbidities, negative controls, study outcomes, and other prognostic factors among fully vaccinated individuals by BNT162b2 and mRNA-1273 who became infected with Covid-19. Data are for individuals fully vaccinated by June 1st, 2021.

	POST-VACCINATION TIME-TO-EVENT Among infected	
Column1	BNT162B26	MRNA-12737
N	5758	3090
Age, mean (SD)	62.66 (18.78)	63.00 (17.14)
18-24, N (%)	128 (2.2)	59 (1.9)
25-29, N (%)	175 (3.0)	71 (2.3)
30-34, N (%)	216 (3.8)	98 (3.2)
35-39, N (%)	263 (4.6)	119 (3.9)
40-44, N (%)	293 (5.1)	165 (5.3)
45-49, N (%)	355 (6.2)	178 (5.8)
50-54, N (%)	466 (8.1)	226 (7.3)
55-59, N (%)	485 (8.4)	241 (7.8)
60-64, N (%)	609 (10.6)	300 (9.7)
65-69, N (%)	513 (8.9)	361 (11.7)
70-74, N (%)	537 (9.3)	425 (13.8)
75-79, N (%)	525 (9.1)	343 (11.1)
80-84, N (%)	433 (7.5)	243 (7.9)
85+, N (%)	760 (13.2)	261 (8.4)
INSURANCE TYPE, N (%)		
MEDICARE ADVANTAGE	2,511 (43.6)	1,505 (48.7)
COMMERCIAL	3,247 (56.4)	1,585 (51.3)
Acquired immune deficiency syndrome (%)	27 (0.5)	25 (0.8)
Alcohol use disorder (%)	111 (1.9)	69 (2.2)
Iron deficiency anemia (%)	1,387 (24.1)	695 (22.5)
Rheumatoid arthritis (%)	377 (6.5)	258 (8.3)
Blood loss anemia (%)	114 (2.0)	70 (2.3)
Congestive heart failure (%)	1,003 (17.4)	484 (15.7)
Chronic obstructive pulmonary disease (%)	1,474 (25.6)	871 (28.2)
Coagulopathy (%)	356 (6.2)	185 (6.0)
Depression (%)	1,485 (25.8)	628 (20.3)
Diabetes without chronic complication (%)	1,561 (27.1)	926 (30.0)
Diabetes with chronic complication (%)	1,282 (22.3)	763 (24.7)
Substance use disorder (%)	285 (4.9)	98 (3.2)
Hypertension (%)	3,397 (59.0)	1,869 (60.5)
Hypothyroidism (%)	1,073 (18.6)	595 (19.3)
Liver disease (%)	402 (7.0)	262 (8.5)
Lymphoma (%)	89 (1.5)	43 (1.4)
Fluid & electrolyte disorder (%)	1,357 (23.6)	735 (23.8)
Metastatic cancer (%)	83 (1.4)	39 (1.3)

Neurological disorder (%)	1,311 (22.8)	478 (15.5)
Obesity (%)	1,480 (25.7)	860 (27.8)
Paralysis (%)	360 (6.3)	103 (3.3)
Peripheral vascular disease (%)	1,321 (22.9)	584 (18.9)
Psychosis (%)	556 (9.7)	180 (5.8)
Pulmonary circulation disorder (%)	213 (3.7)	122 (3.9)
Chronic kidney disease (%)	960 (16.7)	537 (17.4)
Solid tumor without metastasis (%)	434 (7.5)	269 (8.7)
Peptic ulcer disease (%)	57 (1.0)	43 (1.4)
Valvular disorder (%)	636 (11.0)	405 (13.1)
Weight loss (%)	583 (10.1)	208 (6.7)
Stroke cerebrovascular (%)	930 (16.2)	462 (15.0)
Down syndrome (%)	2 (0.0)	0 (0.0)
Thalassemia (%)	11 (0.2)	8 (0.3)
Smoking (%)	461 (8.0)	286 (9.3)
Transplant (%)	10 (0.2)	7 (0.2)
Elixhauser mortality score, mean (SD)	6.50 (10.01)	5.97 (9.19)
Elixhauser readmission score, mean (SD)	20.46 (23.48)	18.74 (21.19)
Transferred from nursing facility/SNF (%)	458 (8.0)	120 (3.9)
Immunologic Rx (%)	2,361 (41.0)	1,467 (47.5)
Immunologic dx (%)	2,034 (35.3)	1,214 (39.3)
Sex		
FEMALE (%)	3,622 (62.9)	1,802 (58.3)
MALE (%)	2,136 (37.1)	1,288 (41.7)
Residence by region (%)		
MIDWEST	12,856 (32.2)	867 (28.1)
NORTHEAST	1,275 (22.1)	546 (17.7)
SOUTH	1,547 (26.9)	994 (32.2)
WEST	1,080 (18.8)	683 (22.1)
Residence Class (%)		
RURAL	1,037 (18.0)	806 (26.1)
URBAN	2,385 (41.4)	1,218 (39.4)
SEMI-URBAN	2,336 (40.6)	1,066 (34.5)
First dose administered before Feb 1, 2021 (%)	3,038 (52.8)	1,424 (46.1)
SES index, mean (SD)	53.24 (2.90)	52.59 (3.25)
Prior any Covid dx (ER/IP/ICU) (%)	3,835 (66.6)	1,805 (58.4)
Prior positive PCR (%)	409 (7.1)	261 (8.4)
Negative outcomes, N (%)		
BILATERAL PRIM OSTEOARTHRITIS KNEE	150 (2.6)	108 (3.5)
Dry eye syndrome	0 (0.0)	0 (0.0)
FREQUENCY OF MICTURITION	215 (3.7)	144 (4.7)
PRESBYOPIA	186 (3.2)	132 (4.3)
Seborrheic keratosis	0 (0.0)	0 (0.0)
SENSORINEURAL HEAR LOSS BILATERAL	160 (2.8)	97 (3.1)
TINEA UNGUIUM	937 (16.3)	295 (9.5)
UTI SITE NOT SPECIFIED	871 (15.1)	376 (12.2)
AGEREL NUCLEAR CATARACT BILATERAL	335 (5.8)	222 (7.2)

	POST-VACCINATION TIME-TO-EVENT AMONG INFECTED	
	BNT162B2	MRNA-1273
N	5758	3090
Age, mean (SD)	62.66 (18.78)	63.00 (17.14)
18-24, N (%)	128 (2.2)	59 (1.9)
25-29, N (%)	175 (3.0)	71 (2.3)

30-34, N (%)	216 (3.8)	98 (3.2)
35-39, N (%)	263 (4.6)	119 (3.9)
40-44, N (%)	293 (5.1)	165 (5.3)
45-49, N (%)	355 (6.2)	178 (5.8)
50-54, N (%)	466 (8.1)	226 (7.3)
55-59, N (%)	485 (8.4)	241 (7.8)
60-64, N (%)	609 (10.6)	300 (9.7)
65-69, N (%)	513 (8.9)	361 (11.7)
70-74, N (%)	537 (9.3)	425 (13.8)
75-79, N (%)	525 (9.1)	343 (11.1)
80-84, N (%)	433 (7.5)	243 (7.9)
85+, N (%)	760 (13.2)	261 (8.4)
INSURANCE TYPE, N (%)		
MEDICARE ADVANTAGE	2,511 (43.6)	1,505 (48.7)
COMMERCIAL	3,247 (56.4)	1,585 (51.3)
Acquired immune deficiency syndrome (%)	27 (0.5)	25 (0.8)
Alcohol use disorder (%)	111 (1.9)	69 (2.2)
Iron deficiency anemia (%)	1,387 (24.1)	695 (22.5)
Rheumatoid arthritis (%)	377 (6.5)	258 (8.3)
Blood loss anemia (%)	114 (2.0)	70 (2.3)
Congestive heart failure (%)	1,003 (17.4)	484 (15.7)
Chronic obstructive pulmonary disease (%)	1,474 (25.6)	871 (28.2)
Coagulopathy (%)	356 (6.2)	185 (6.0)
Depression (%)	1,485 (25.8)	628 (20.3)
Diabetes without chronic complication (%)	1,561 (27.1)	926 (30.0)
Diabetes with chronic complication (%)	1,282 (22.3)	763 (24.7)
Substance use disorder (%)	285 (4.9)	98 (3.2)
Hypertension (%)	3,397 (59.0)	1,869 (60.5)
Hypothyroidism (%)	1,073 (18.6)	595 (19.3)
Liver disease (%)	402 (7.0)	262 (8.5)
Lymphoma (%)	89 (1.5)	43 (1.4)
Fluid & electrolyte disorder (%)	1,357 (23.6)	735 (23.8)
Metastatic cancer (%)	83 (1.4)	39 (1.3)
Neurological disorder (%)	1,311 (22.8)	478 (15.5)
Obesity (%)	1,480 (25.7)	860 (27.8)
Paralysis (%)	360 (6.3)	103 (3.3)
Peripheral vascular disease (%)	1,321 (22.9)	584 (18.9)
Psychosis (%)	556 (9.7)	180 (5.8)
Pulmonary circulation disorder (%)	213 (3.7)	122 (3.9)
Chronic kidney disease (%)	960 (16.7)	537 (17.4)
Solid tumor without metastasis (%)	434 (7.5)	269 (8.7)
Peptic ulcer disease (%)	57 (1.0)	43 (1.4)
Valvular disorder (%)	636 (11.0)	405 (13.1)
Weight loss (%)	583 (10.1)	208 (6.7)
Stroke cerebrovascular (%)	930 (16.2)	462 (15.0)
Down syndrome (%)	2 (0.0)	0 (0.0)
Thalassemia (%)	11 (0.2)	8 (0.3)
Smoking (%)	461 (8.0)	286 (9.3)
Transplant (%)	10 (0.2)	7 (0.2)
Elixhauser mortality score, mean (SD)	6.50 (10.01)	5.97 (9.19)
Elixhauser readmission score, mean (SD)	20.46 (23.48)	18.74 (21.19)
Transferred from nursing facility/SNF (%)	458 (8.0)	120 (3.9)
Immunologic Rx (%)	2,361 (41.0)	1,467 (47.5)
Immunologic dx (%)	2,034 (35.3)	1,214 (39.3)

Sex		
FEMALE (%)	3,622 (62.9)	1,802 (58.3)
MALE (%)	2,136 (37.1)	1,288 (41.7)
Residence by region (%)		
MIDWEST	1,856 (32.2)	867 (28.1)
NORTHEAST	1,275 (22.1)	546 (17.7)
SOUTH	1,547 (26.9)	994 (32.2)
WEST	1,080 (18.8)	683 (22.1)
Residence Class (%)		
RURAL	1,037 (18.0)	806 (26.1)
URBAN	2,385 (41.4)	1,218 (39.4)
SEMI-URBAN	2,336 (40.6)	1,066 (34.5)
First dose administered before Feb 1, 2021 (%)	3,038 (52.8)	1,424 (46.1)
SES index, mean (SD)	53.24 (2.90)	52.59 (3.25)
Prior any Covid dx (ER/IP/ICU) (%)	3835 (66.6)	1,805 (58.4)
Prior positive PCR (%)	409 (7.1)	261 (8.4)
Negative outcomes, N (%)		
BILATERAL PRIM OSTEOARTHRITIS KNEE	150 (2.6)	108 (3.5)
Dry eye syndrome	0 (0.0)	0 (0.0)
FREQUENCY OF MICTURITION	215 (3.7)	144 (4.7)
PRESBYOPIA	186 (3.2)	132 (4.3)
Seborrheic keratosis	0 (0.0)	0 (0.0)
SENSORINEURAL HEAR LOSS BILATERAL	160 (2.8)	97 (3.1)
TINEA UNGUIUM	937 (16.3)	295 (9.5)
UTI SITE NOT SPECIFIED	871 (15.1)	376 (12.2)
AGEREL NUCLEAR CATARACT BILATERAL	335 (5.8)	222 (7.2)

Figure S12. Time to event for hospitalization/ ICU/ death/ transfer to hospice.
Kaplan-Meier curves for time-to-events without truncation for the event of (A) hospitalization/ ICU/death/transfer to hospice whichever occurs first among infected patients, and (B) ICU/death/ transfer hospice whichever occurs first among infected patients.

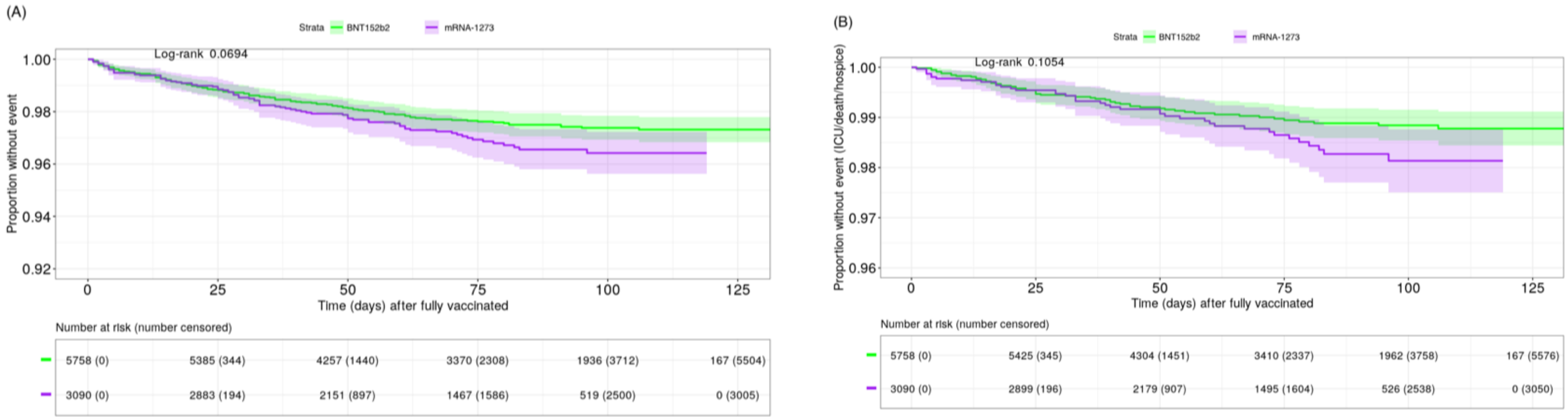

Table S7. Standardized mean differences of covariates in unweighted and weighted (IPTW) samples between BNT162b2 and mRNA-1273 vaccinated individuals.

	POST-VACCINATION TIME-TO-EVENT among infected individuals	
Column1	Unweighted	IPTW
Age†¶‡	0.019	0.044
Insurance Type†¶‡	0.102	0.054
Acquired immune deficiency syndrome†‡	0.043	0.002
Alcohol abuse†‡	0.021	0.002
Iron deficiency anemia†‡	0.038	0.028
Rheumatoid arthritis†‡	0.069	0.001
Blood loss anemia†‡	0.02	0.009
Congestive heart failure†‡	0.047	0.02
Chronic obstructive pulmonary disease†‡	0.058	0.007
Coagulopathy†‡	0.008	0.006
Depression†‡	0.13	0.033
Diabetes without chronic complication†‡	0.063	0.007
Diabetes with chronic complication†‡	0.057	0.01
Substance use disorder†	0.09	0.04
Hypertension†‡	0.03	0.022
Hypothyroidism†‡	0.016	0.02
Liver disease†‡	0.056	0.001
Lymphoma†‡	0.013	0.014
Fluid & electrolyte disorder†‡	0.005	0.007
Metastatic cancer†‡	0.016	0.011
Neurological disorder†‡	0.186	0.054
Obesity†‡	0.048	0.012
Paralysis†‡	0.137	0.042
Peripheral vascular disease†‡	0.1	0.046
Psychosis†‡	0.144	0.026
Pulmonary circulation disorder†‡	0.013	0.004
Chronic kidney disease†‡	0.019	0.019
Solid tumor without metastasis†‡	0.043	<0.001
Peptic ulcer disease†‡	0.037	0.004
Valvular disorder†‡	0.063	0.003
Weight loss†‡	0.122	0.036
Stroke cerebrovascular†	0.033	0.032
Down syndrome†	0.026	0.029
Thalassemia†	0.014	0.003
Smoking†	0.045	0.009
Transplant†	0.012	0.002
Elixhauser mortality score	0.055	0.027
Elixhauser readmission score	0.077	0.044
Nursing facility/SNF†‡	0.173	0.068
Immunologic Rx	0.131	0.062
Immunologic Dx†‡	0.082	0.013
Sex†‡	0.094	0.021
Residence by state†¶‡	0.548	0.072
Residence by region*	0.176	0.011
First dose administered before February 1, 2021†¶*	0.134	0.058
SES index†¶‡	0.209	0.02
Prior any Covid Dx (ER/IP/ICU/Dx) †¶	0.17	0.035
Prior positive PCR	0.05	0.062
Urban/ Rural residence†¶‡	0.202	0.019

	POST-VACCINATION TIME-TO-EVENT among infected individuals	
	Unweighted	IPTW
Age†¶‡	0.019	0.044
Insurance Type†¶‡	0.102	0.054
Acquired immune deficiency syndrome†‡	0.043	0.002
Alcohol abuse†‡	0.021	0.002
Iron deficiency anemia†‡	0.038	0.028
Rheumatoid arthritis†‡	0.069	0.001
Blood loss anemia†‡	0.02	0.009
Congestive heart failure†‡	0.047	0.02
Chronic obstructive pulmonary disease†‡	0.058	0.007
Coagulopathy†‡	0.008	0.006
Depression†‡	0.13	0.033
Diabetes without chronic complication†‡	0.063	0.007
Diabetes with chronic complication†‡	0.057	0.01
Substance use disorder†	0.09	0.04
Hypertension†‡	0.03	0.022
Hypothyroidism†‡	0.016	0.02
Liver disease†‡	0.056	0.001
Lymphoma†‡	0.013	0.014
Fluid & electrolyte disorder†‡	0.005	0.007
Metastatic cancer†‡	0.016	0.011
Neurological disorder†‡	0.186	0.054
Obesity†‡	0.048	0.012
Paralysis†‡	0.137	0.042
Peripheral vascular disease†‡	0.1	0.046
Psychosis†‡	0.144	0.026
Pulmonary circulation disorder†‡	0.013	0.004
Chronic kidney disease†‡	0.019	0.019
Solid tumor without metastasis†‡	0.043	<0.001
Peptic ulcer disease†‡	0.037	0.004
Valvular disorder†‡	0.063	0.003
Weight loss†‡	0.122	0.036
Stroke cerebrovascular†	0.033	0.032
Down syndrome†	0.026	0.029
Thalassemia†	0.014	0.003
Smoking†	0.045	0.009
Transplant†	0.012	0.002
Elixhauser mortality score	0.055	0.027
Elixhauser readmission score	0.077	0.044
Nursing facility/SNF†‡	0.173	0.068
Immunologic Rx	0.131	0.062
Immunologic Dx†‡	0.082	0.013
Sex†‡	0.094	0.021
Residence by state†¶‡	0.548	0.072
Residence by region*	0.176	0.011
First dose administered before February 1, 2021†¶*	0.134	0.058
SES index†¶‡	0.209	0.02

Prior any Covid Dx (ER/IP/ICU/Dx) †¶	0.17	0.035
Prior positive PCR	0.05	0.062
Urban/ Rural residence†¶‡	0.202	0.019

[†]Adjusted in propensity score model. Variables are adjusted in outcome regression models (as in equation SM 7-8).
[¶‡]Variables are adjusted in outcome regression models (as in equation SM 4-6).
[*]Residence by region is used instead of residence by state in Cox-PH model.
[‡]Variables are adjusted in negative outcome regression models (as in equation SM 4-6).

Figure S13. Incidence of events 10d, 30d, 50d, 70d, and 90d post-vaccination among vaccinated and infected individuals. Adjusted hazard ratios (aHRs) along with 95% confidence intervals, number needed to treat (NNT), and predicted marginal rates per 1,000 individuals (in parenthesis) of experiencing events at 10d, 30d, 50d, 70d, and 90d post vaccination (last dose + 14d) for mRNA-1273 (Moderna) and BNT162b2 (Pfizer), respectively (separated by “/”). Time-to-event for adverse outcomes (top-panel) ICU/death/hospice and (bottom-panel) hospitalization/ICU/death/hospice whichever occurring first among infected-vaccinated individuals.

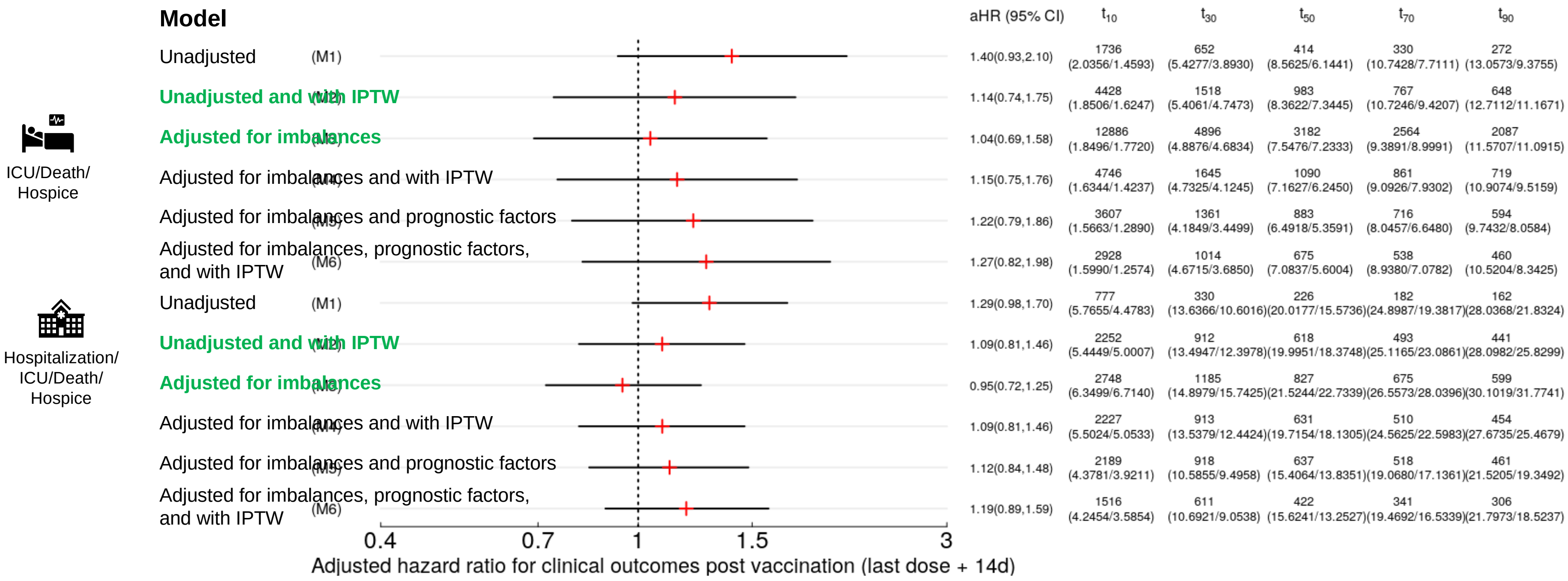

aHR (95% CI)	t_{10}	t_{30}	t_{50}	t_{70}	t_{90}
1.40(0.93,2.10)	1736 (2.0356/1.4593)	652 (5.4277/3.8930)	414 (8.5625/6.1441)	330 (10.7428/7.7111)	272 (13.0573/9.3755)
1.14(0.74,1.75)	4428 (1.8506/1.6247)	1518 (5.4061/4.7473)	983 (8.3622/7.3445)	767 (10.7246/9.4207)	648 (12.7112/11.1671)
1.04(0.69,1.58)	12886 (1.8496/1.7720)	4896 (4.8876/4.6834)	3182 (7.5476/7.2333)	2564 (9.3891/8.9991)	2087 (11.5707/11.0915)
1.15(0.75,1.76)	4746 (1.6344/1.4237)	1645 (4.7325/4.1245)	1090 (7.1627/6.2450)	861 (9.0926/7.9302)	719 (10.9074/9.5159)
1.22(0.79,1.86)	3607 (1.5663/1.2890)	1361 (4.1849/3.4499)	883 (6.4918/5.3591)	716 (8.0457/6.6480)	594 (9.7432/8.0584)
1.27(0.82,1.98)	2928 (1.5990/1.2574)	1014 (4.6715/3.6850)	675 (7.0837/5.6004)	538 (8.9380/7.0782)	460 (10.5204/8.3425)
1.29(0.98,1.70)	777 (5.7655/4.4783)	330 (13.6366/10.6016)	226 (20.0177/15.5736)	182 (24.8987/19.3817)	162 (28.0368/21.8324)
1.09(0.81,1.46)	2252 (5.4449/5.0007)	912 (13.4947/12.3978)	618 (19.9951/18.3748)	493 (25.1165/23.0861)	441 (28.0982/25.8299)
0.95(0.72,1.25)	2748 (6.3499/6.7140)	1185 (14.8979/15.7425)	827 (21.5244/22.7339)	675 (26.5573/28.0396)	599 (30.1019/31.7741)
1.09(0.81,1.46)	2227 (5.5024/5.0533)	913 (13.5379/12.4424)	631 (19.7154/18.1305)	510 (24.5625/22.5983)	454 (27.6735/25.4679)
1.12(0.84,1.48)	2189 (4.3781/3.9211)	918 (10.5855/9.4958)	637 (15.4064/13.8351)	518 (19.0680/17.1361)	461 (21.5205/19.3492)
1.19(0.89,1.59)	1516 (4.2454/3.5854)	611 (10.6921/9.0538)	422 (15.6241/13.2527)	341 (19.4692/16.5339)	306 (21.7973/18.5237)

Figure S14. Predicted survival probabilities.
Predicted survival probabilities based on the univariate model with IPTW for (A) ICU/death/hospice and (B) hospitalization/ICU/death/hospice whichever occurring first among infected individuals. Predicted survival probabilities of a typical (prediction at mode) infected patient based on the adjusted model for imbalances along with IPTW with respect to (C) ICU/death/hospice and (D) hospitalization/ICU/death/hospice whichever occurs first.

Time-to-ICU, death, or discharge to hospice	Time-to-hospitalization, ICU, death, or discharge to hospice

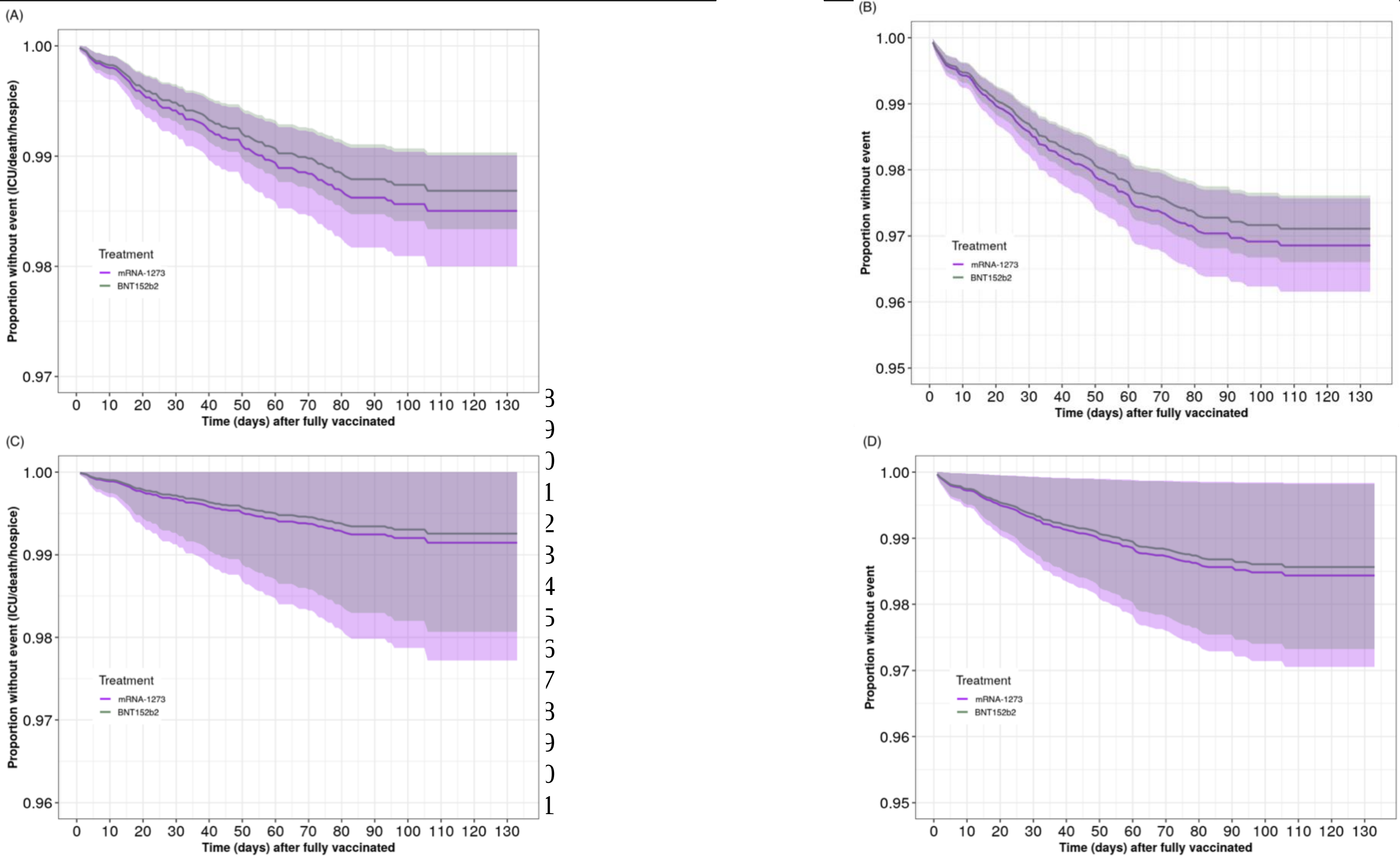

Table S8. Summary tables for responses by time-period which roughly correspond to early-, pre-alpha, and alpha-dominant periods of the pandemic.

Study-related outcomes	*Before February 28*		*March 1–March 31, 2021*		*April 1–May 1, 2021*	
	BNT162B2	MRNA-1273	BNT162B2	MRNA-1273	BNT162B2	MRNA-1273
Fully Vaccinated	199069	149466	361072	415817	820430	493420
Infection; N (%)	1659 (0.8)	454 (0.3)	901 (0.2)	753 (0.2)	1206 (0.1)	742 (0.2)
Hospitalization/ICU/ deceased/ transferred to hospice; N (%)	35 (0.0)	7 (0.0)	15 (0.0)	19 (0.0)	22 (0.0)	16 (0.0)
ICU/deceased/ transferred to hospice; N (%)	17 (0.0)	1 (0.0)	5 (0.0)	8 (0.0)	10 (0.0)	5 (0.0)
Hospitalization (%)	30 (0.0)	6 (0.0)	11 (0.0)	16 (0.0)	17 (0.0)	13 (0.0)
ICU; N (%)	14 (0.0)	1 (0.0)	5 (0.0)	8 (0.0)	9 (0.0)	5 (0.0)
Deceased/ transferred to hospice; N (%)	7 (0.0)	0 (0.0)	0 (0.0)	1 (0.0)	2 (0.0)	1 (0.0)

Supplement 3

Methods

Let i denote vaccinated patients such that $i = 1, \ldots, N$ and $N_1 + N_2 = N$ where N_1 and N_2 are the total number of subjects vaccinated by mRNA-1273 (Moderna) and BNT162b2 (Pfizer), respectively. Let Y_i refer to a Covid-related adverse outcome recorded for the i-th patient taking value of 1 if a patient experiences an event post vaccination date (last dose + 14day) or 0, otherwise. Here Y_i corresponds to (a) infection (associated with Covid diagnoses, hospitalization, intensive care unit (ICU) or emergency (ER) visit, mortality, and/or hospice-transfer whichever occurring first), (b) hospitalization, (c) composite event consisting of ICU, mortality, and transfer-to-hospice whichever occurring first and (d) composite event exploiting hospitalization, ICU, mortality, and transfer-to-hospice whichever occurring first. Denote by U_{im} the m-th socio-demographic variable (e.g., age, gender, socio-economic status (SES), residence of state) and X_{il} the l-th comorbidity status (Elixhauser-based comorbidities, CDC reported risk factors that are not included in the Elixhauser readmission or mortality score (e.g., smoking)) associated with the i-th subject. In addition, we consider Covid specific covariates (i.e., timing of vaccination and prior history of Covid diagnoses) which are denoted by T_{ir}. Define by U_i, X_i, and T_i as $M, L,$ and R dimensional vector of covariates for the i-the subject; where $M = |M_0|$, $L = |L_0|$, and $R = |R_0|$ are the cardinals of sets, $M_0, L_0,$ and R_0. Let V_i be a binary variable indicating the type of vaccines administered taking value 1 if a subject takes mRNA-1273 and 0 if a subject receives BNT162b2 vaccine within the recommended CDC time-interval (4 days earlier and 28 days later from the recommended second dose CITE CDC) between the first and second doses. The primary objective of this study is to assess the relative vaccine effectiveness between these two treatment arms with respect to morbidity and mortality. We use both logistic regression and Cox-proportional hazard (PH) model assuming right censoring. In contrast to logistic regression, Cox-PH exploits data available until May 31, 2021; see below. For logistic models, we truncate samples with respect to complete vaccination date (2nd dose + 14 days) on May 1, 2021, April 1, 2021, and March 2, 2021, for 30-day, 60-day, and 90-day outcome regression so that each subject had at-least 30-, 60-, and 90-day window of follow-up period to experience events, respectively. For time-to-event data, we use June 1, 2021, as a censoring date. In the outcome regression, we categorize numeric variables (age, SES index, and timing of vaccines) to accommodate nonlinear relationship between the corresponding variable and the response,

A.1 Multivariable generalized linear model for outcome regression

We cast the following multivariable generalized linear model (GLM) to characterize the association between covariates and binary outcome.

1. $\text{logit} \Pr(Y_i = 1) = \alpha + \sum_{m=1}^{M} U_{im}\delta_m + \sum_{l=1}^{L} X_{il}\beta_l + \sum_{r=1}^{R} T_{ir}\tau_r + V_i\theta.$

Here α, $\delta_m, \beta_l, \tau_r,$ and θ correspond to the fixed parameters associated with intercept, socio-demographic, comorbidities, Covid-specific variables, and vaccination type, respectively. Note the above model is a saturated model and we fit a reduced model having a varying set of covariates as below with and without IPTW to assess how the inference of θ changes under different model specifications and settings. With an abuse of notation, let Y_i denote the binary response of interest in both unweighted and weighted samples. Denote by M_1 and R_1 the set of baseline characteristics related to socio-demographic variables (age, SES index, residence by

state or region) and Covid-related features (e.g., timing of vaccination, prior history of Covid diagnoses) that are imbalanced between two treatment arms; such that $M_1 \subseteq M_0$ and $R_1 \subseteq R_0$. Denote by $M_2, L_2,$ and R_2 the set of covariates that are associated with the corresponding response of interests and are chosen empirically via univariate association filter approach to screen noise variables. Note a more formal approach based on a regularization (sparse) penalty may also be adopted; however, if such approach is used, the post-selection inference[1] is warranted. Here we fitted a series of univariate GLMs with a logit link function corresponding to each variable of interest based on the unweighted sample. We performed hypothesis tests for null effects via Wald test statistics, compared p-values, and selected the ones that are less than a prespecified threshold of 0.10. Let $M_2 \subseteq M_0, L_2 \subseteq M_0$ and $R_2 \subseteq R_0$. We fit GLM(s)[2] using the logit link function and estimate the parameters by the iteratively re-weighted least square (IRLS) algorithm; standard errors are estimated via robust, sandwich covariance estimator to account for the fact that weights are estimated and exploited.

		Adjustment	IPTW	Subclassification
(SM1)	$\text{logit}\Pr(Y_i = 1) = \alpha + V_i\theta$	X	X	X
(SM2)	$\text{logit}\Pr(Y_i = 1) = \alpha + V_i\theta$	X	X	X
(SM3)	$\text{logit}\Pr(Y_{ik} = 1) = \alpha_k + V_{ik}\theta_k$	X	X	X
(SM4)	$\text{logit}\Pr(Y_i = 1) = \alpha + \sum_{m \in M_1} U_{im}\delta_m + \sum_{r \in R_1} T_{ir}\tau_r + V_i\theta$	X	X	X
(SM5)	$\text{logit}\Pr(Y_i = 1) = \alpha + \sum_{m \in M_1} U_{im}\delta_m + \sum_{r \in R_1} T_{ir}\tau_r + V_i\theta$	X	X	X
(SM6)	$\text{logit}\Pr(Y_{ik} = 1) = \alpha_k + \sum_{m \in M_1} U_{ikm}\delta_{mk} + \sum_{r \in R_1} T_{ikr}\tau_{rk} + V_{ik}\theta_k$	X	X	X
(SM7)	$\text{logit}\Pr(Y_i = 1) = \alpha + \sum_{m \in M_2} U_{im}\delta_m + \sum_{l \in L_2} X_{il}\beta_l + \sum_{r \in R_2} T_{ir}\tau_r + V_i\theta.$	X	X	X
(SM8)	$\text{logit}\Pr(Y_i = 1) = \alpha + \sum_{m \in M_2} U_{im}\delta_m + \sum_{l \in L_2} X_{il}\beta_l + \sum_{r \in R_2} T_{ir}\tau_r + V_i\theta.$	X	X	X

A.2 Multivariable Cox-PH model for time-to-event outcome regression

In contrast to the discrete study endpoints analyses, we considered the time to the occurrence of a Covid-related event across the study period assuming right censoring. In this pursuit, we fitted

semi-parametric Cox-PH model. Let t_i be the event time for the i-th subject and C_i be the censoring time. Let $h(t|U_i, X_i, T_i, V_i)$ be the hazard rate for an individual at some time t and $h_0(t) > 0$ be the non-parametric part of the model signaling the baseline hazard function at the population-level. Cox-PH model assumes

$$2.\quad h(t|U_i, X_i, T_i, V_i) = h_0(t)e^{\sum_{m=1}^{M} U_{im}\delta_m + \sum_{l=1}^{L} X_{il}\beta_l + \sum_{r=1}^{R} T_{ir}\tau_r + V_i\theta};$$

the definition of all other terms remains as same as before. Using the similar intuition as above, we screen variables based on a series of univariate Cox-PH models where the nullity of effects is assessed by likelihood ratio test. We observed superior screening performance, especially for the variables with less cell frequencies, using the LRT approach relative to Wald based test statistic. We fit the univariate-and-multivariable models (similar to SM1-SM8) with and without adjustments by inverse probability of treatment weighting that is to be discussed in B.1. We estimate the parameters by maximizing the partial likelihood function and standard errors are estimated via robust, sandwich covariance estimator. Reported are the adjusted hazard ratios (aHRs) along with 95% CIs.

B. Estimation of propensity scores and covariate balances

Consider the following generalized linear model with log odds of receiving mRNA-1273. A logit link for the binary vaccine type depicting the conditional probability of receiving mRNA-1273 is used. Denote by $\alpha_{(p)}, \delta_{m(p)}, \beta_{l(p)}, and\ \tau_{r(p)}$ the regression parameters quantifying the association between vaccine type and observed covariates; the subscript '(p)' is used to differentiate the parameters of the propensity model from the outcome model.

$$3.\quad \text{logit}\Pr(V_i = 1) = \alpha_{(p)} + \sum_{m=1}^{M} U_{im}\delta_{m(p)} + \sum_{l=1}^{L} X_{il}\,\beta_{l(p)} + \sum_{r=1}^{R} T_{ir}\tau_{r(p)}$$

$$\Pr(V_i = 1|U_i, X_i, T_i) = \frac{1}{1 + \exp\left(-\alpha_{(p)} - \sum_{m=1}^{M} U_{im}\delta_{m(p)} - \sum_{l=1}^{L} X_{il}\,\beta_{l(p)} - \sum_{r=1}^{R} T_{ir}\tau_{r(p)}\right)}$$

Let $\Pr(V_i = 1|U_i = u_i, X_i = x_i, T_i = t_i) = e(u_i, x_i, t_i)$ be the propensity score of the i-th subject. For the notational convenience, assume $e(u_i, x_i, t_i) = e_i$.

B.1. Inverse probability of treatment weighting (IPTW)

We used inverse probability of treatment assignment as a weight in the multivariate outcome analyses; the main purpose of using IPTW analyses is to create a weighted sample in which the distribution of either the measured confounding variables or the prognostically important covariates is same between vaccinated people with respect to two treatment arms. Such propensity score based weighting approach takes a differential amount of information from each subject depending on one's conditional likelihood of receiving mRNA-1273. This weighted method corrects and reduces the magnitude of selection bias balancing the observed differences between treatments (i.e., mRNA-1273 and BNT162b2 vaccinated people in our application) with respect to different risk factors in the sample.

Define the corresponding subject's IPTW by $w_i = \frac{V_i}{e_i} + \frac{1-V_i}{1-e_i}$. Note that vaccinated subjects with mRNA-1273 having a very low propensity score (close to zero) can result in a very large weight; similarly, a BNT-162b2 vaccinated subject with a very high propensity score (close to one) can result in a very large weight; such instability may increase variability of the estimated treatment effect. As a remedy, the stabilized weights $w_i = \frac{\Pr(V_i=1)V_i}{e_i} + \frac{\Pr(V_i=0)(1-V_i)}{1-e_i}$ are adopted; where $\Pr(V_i = 1)$ and $\Pr(V_i = 0)$ denote the marginal probabilities estimated as

$$4. \quad \frac{\sum_{i\in N_1} e_i}{N_1} \text{ and } \frac{\sum_{i\in N_2}(1-e_i)}{N_2},$$

for mRNA-1273 and BNT162b2, respectively. We convert marginal probabilities to marginal rates per 1,000 individuals by multiplying the marginal probabilities above by 1,000.

Another alternative to address the problems arising from very large weights is to use trimmed or truncated weights; where we trim extreme probability values (i.e., less than 0.01 and greater than 0.99) and replace them with prespecified values such as 0.01 and 0.99, respectively.[3,4] Propensity score-based weighting is analogous to a weighted analysis that treats IPTWs, w_i's, as sampling weights; applying these weights to the study subjects creates a synthetic sample which tends to balance all covariates (i.e., U_i, X_i, T_i) used to estimate the weights. Here each subject, say the i-th subject, amounts to a total of w_i subjects resulting in a total of $\sum_i w_i$ subjects. The sampling weights are specified in each outcome regression analysis specified in A.1 and A.2. Fixed parameters are estimated by optimizing the corresponding weighted target function (e.g., (partial) log likelihood function) where each subject's contribution is weighted by w_i's. Estimation of standard errors accounts for the weighted nature of the synthetic sample via robust, sandwich covariance estimators. Implementation of such models is straightforward as most software packages allow to specify the name of a weight variable in procedures of multivariable analyses. Prior to running weighted-outcome analyses, balance on observed covariates are also checked via standardized mean and weighted regression which are discussed in section B.3.

B.2. Propensity score subclassification

We divide the sample into K strata using quartiles or quintiles of the estimated propensity scores, e_i and evaluate the relative treatment effectiveness in terms of aOR or RR within each stratum by running univariate and multivariable GLM. Let aOR for treatment effect be θ_k at the k-th stratum. We aggregate the K estimates by using the weighted average and obtain the corresponding weighted standard error by aggregating variances over all strata; here weights being the total number of samples within each stratum. We check balances of covariates within each stratum in terms of SMD.

B.3. Enumerating covariate balances

We computed the standardized (mean) difference (SMD) as a measure of distance between two treatment groups in terms of each potential risk factors; this can be viewed as a measure of balance for each covariate before and after propensity score adjustments to ensure that weighting helps correct selection bias. A SMD value greater than 0.10 signals substantial difference between two treatment arms in terms of the corresponding covariate. We computed SMD for weighted data following Austin et al. (2015).[5]

In addition, we also performed a statistical significance testing approach based on univariate weighted regression. Here we run a weighted simple linear regression using a continuous covariate (e.g., age) as the dependent variable and V_i as the single independent variable with weight, w_i. Similarly, we run a weighted simple logistic regression for binary covariate and weighted multinomial regression for a covariate with more than two classes (e.g., residential state). We used Wald test statistics with standard error estimated by robust, sandwich covariance estimator. We acknowledge that failing to reject a null hypothesis in any test does not necessarily indicate balances on the sample as this procedure indeed tests about the nullity of the population parameter whereas we are more interested in inducing balances only on the analytical sample.

C. Marginal effects estimation

The mean predicted marginal probabilities of experiencing events by mRNA-1273 and BNT162b2 are calculated via “recycled predictions” based on each model.[6,7] This approach is also known as marginal standardization in the literature where the idea is to calculate the marginal effect (instantaneous effect on the predicted probability of response due to a change from one vaccine to the other while keeping other variables constant) at each individual level and then calculate the sample average of individual marginal effects to obtain the overall marginal effect. Let i^* and i^{**} refer to subjects each, hypothetically, having mRNA-1273 and BNT162b2, respectively. This follows that

5. $$p_{i,mRNA} = \Pr(Y_{i^*} = 1 | U_i, X_i, T_i, V_{i^*}) = \frac{1}{1+\exp(-\alpha - \sum_{m \in M_2} U_{im}\delta_m - \sum_{l \in L_2} X_{il}\beta_l - \sum_{r \in R_2} T_{ir}\tau_r - V_{i^*}\theta)}$$

6. $$p_{i,BNT} = \Pr(Y_{i^*} = 1 | U_i, X_i, T_i, V_{i^{**}}) = \frac{1}{1+\exp(-\alpha - \sum_{m \in M_2} U_{im}\delta_m - \sum_{l \in L_2} X_{il}\beta_l - \sum_{r \in R_2} T_{ir}\tau_r)}$$

The mean predicted marginal probabilities (risk) for mRNA-1273 and BNT162b2 are calculated as
$p_{mRNA} = \sum_{i=1}^{N} p_{i,mRNA} / N$ and $p_{BNT} = \sum_{i=1}^{N} p_{i,BNT} / N$, respectively. Number needed to treat is calculated as a reciprocal of the absolute risk difference, $|p_{mRNA} - p_{BNT}|$.[8,9]

D. Model diagnostics

We assess the numerical performances of logistic regression by McFadden, Craig-Uhler’s pseudo R-squared values, Somer’s D_{xy}, and C-statistic. Table S8 provides the summary measures for the model (SM8). In general, as we add more clinically relevant covariates, the model quantifies more variation in response reflected in better predictive performance. Collinearity between covariates is assessed by generalized variance inflation factor (GVIF); where all values are less than 4.00. The proportionality assumption for Cox-PH model is validated visually by plotting Schoenfeld residuals versus time and hypothesis testing.

Table S8. Diagnostics of multivariable propensity model with respect to datasets based on different selection criteria.

	Model	C-Statistic	Somer's D_{xy}	Pseudo R-squared McFadden (Craig-Uhler)
**A**	Propensity model for day-30 (N = 2,439,274)	0.64	0.28	0.04 (0.08)
**B**	Propensity model for day-60 (N = 1,153,411)	0.62	0.25	0.04 (0.06)
**C**	Propensity model for day-90 (N = 386,930)	0.66	0.33	0.06 (0.11)
**D**	Propensity model for time-to-adverse-event among infected (N = 8,848)	0.69	0.37	0.08 (0.14)
**E**	Propensity model for time-to-adverse-event for a subset of population without any prior COVID diagnoses before last dose (N = 3,686,087)	0.63	0.27	0.07 (0.04)
**F**	Propensity model for time-to-adverse-event for a subset of population having prior COVID diagnoses before first dose (N = 265,450)	0.62	0.25	0.07 (0.04)
**G**	Propensity model for time-to-adverse-event for a subset of population aged greater than or equal to 65 (N = 1,084,067)	0.64	0.28	0.05 (0.08)
**H**	Propensity model for time-to-adverse-event for a subset of population aged less than 65 (N = 2,878,988)	0.61	0.21	0.03 (0.05)
**I**	Propensity model for time-to-event among all vaccinated (N = 3,963,055)	0.63	0.27	0.04 (0.07)

E. Adjustment for unmeasured confounding and selection bias

As sensitivity analyses for unmeasured confounding, we report "E-values"[10] of adjusted odds and hazard ratios for the vaccine type with respect to different models. Unlike p-values, E-values are not susceptible to large sample size and quantify the smallest strength of association in terms of risk ratio that an unmeasured confounder would need to have with both the treatment and the outcome variable to fully explain away a specific treatment-outcome association, conditional on the measured covariates.

We also adopt negative control approach where we pre-specify binary negative control outcomes within each cohort that are highly unlikely to be associated with Covid vaccines but might have

an underlying association with observed confounding variables (e.g., age) at an extent. We select the list of potential negative outcomes following Khera et al[11] as below:

1. Identify the 500 most common primary diagnosis codes in 2019 affecting individuals in each cohort, ignoring multiple diagnoses for the same condition for the same individual. Repeat for additional cohort.
2. Filter the observed diagnoses to only retain diagnoses observed in both cohorts.
3. Rank the diagnosis codes in each cohort and then sum the ranks for a composite rank sum (e.g., low back pain is #13 in BNT162b2and #15 in mRNA-1273 for a rank sum of 28).
4. Order the diagnosis codes by rank sums from smallest to largest.
5. Take the top 10/20/50/etc. outcomes that meet the criteria for negative control outcomes.

We fit multivariable GLMs with logit link functions with respect to each negative control outcomes adjusting for observed confounding variables excluding the ones that are linked to Covid-19 (e.g., previous history of SARS-CoV-2 infection) except vaccine type. We estimate the null distribution of systematic error and quantify the bias; a mean bias value close to 0 indicates that the analysis is not subject to major concern.[12] While the idea of using a reference set of negative controls to detect whether bias is likely to be a major concern has advantages, the calibration of p-values may be problematic due to the violation of assumptions. For an instance, it is likely that the Covid-19 vaccine-outcome analysis is not subject to the same sources of bias as the set of negative controls and the number of negative control to establish the parameters of the null distribution was not adequate (less than 25); see Gruber et al.[13]

References for Supplement

1. Taylor J, Tibshirani R. Post-selection inference for ℓ1-penalized likelihood models. *Can J Statistics*. **46**, 41–61 (2018). doi:10.1002/cjs.11313

2. McCullagh P, Nelder JA. *Generalized Linear Models*. 2nd ed. Routledge; 2019. doi:10.1201/9780203753736

3. Lee BK, Lessler J, Stuart EA. Weight Trimming and Propensity Score Weighting. Biondi-Zoccai G, ed. *PLoS ONE*. **6**, e18174 (2011). doi:10.1371/journal.pone.0018174

4. Cole SR, Hernan MA. Constructing Inverse Probability Weights for Marginal Structural Models. *American Journal of Epidemiology*. **168**, 656–664 (2008). doi:10.1093/aje/kwn164

5. Austin PC, Stuart EA. Moving towards best practice when using inverse probability of treatment weighting (IPTW) using the propensity score to estimate causal treatment effects in observational studies. *Statist Med*. **34**, 3661–3679 (2015). doi:10.1002/sim.6607

6. Muller CJ, MacLehose RF. Estimating predicted probabilities from logistic regression: different methods correspond to different target populations. *International Journal of Epidemiology*. **43**, 962–970 (2014). doi:10.1093/ije/dyu029

7. Marginal Effects and Adjusted Predictions. In: *SAGE Research Methods Foundations*. SAGE Publications Ltd; 2020. doi:10.4135/9781526421036939917

8. Zhang Z, Ambrogi F, Bokov AF, Gu H, de Beurs E, Eskaf K. Estimate risk difference and number needed to treat in survival analysis. *Ann Transl Med*. **6**, 120 (2018). doi:10.21037/atm.2018.01.36

9. Cook RJ, Sackett DL. The number needed to treat: a clinically useful measure of treatment effect. *BMJ*. **310**, 452–454 (1995). doi:10.1136/bmj.310.6977.452

10. VanderWeele TJ, Ding P. Sensitivity Analysis in Observational Research: Introducing the E-Value. *Ann Intern Med*. **167**, 268 (2017). doi:10.7326/M16-2607

11. Khera R, Clark C, Lu Y, et al. Association of Angiotensin- Converting Enzyme Inhibitors and Angiotensin Receptor Blockers With the Risk of Hospitalization and Death in Hypertensive Patients With COVID- 19. *JAHA*. **10** (2021). doi:10.1161/JAHA.120.018086

12. Schuemie MJ, Ryan PB, DuMouchel W, Suchard MA, Madigan D. Interpreting observational studies: why empirical calibration is needed to correct p- values. *Statist Med*. **33**, 209–218 (2014). doi:10.1002/sim.5925

13. Gruber S, Tchetgen Tchetgen E. Limitations of empirical calibration of p-values using observational data: Limitations of p-value calibration. *Statist Med*. **35**, 3869–3882 (2016). doi:10.1002/sim.6936
