# Supplementary material for: Comparative effectiveness over time of the mRNA-1273 (Moderna) vaccine and the BNT162b2 (Pfizer-BioNTech) vaccine
Source: Nat Commun. 2022 May 2;13:2377. doi: 10.1038/s41467-022-30059-3 (PMC9061778; doi:10.1038/s41467-022-30059-3)
Supplement: Supplementary file 3 — Reporting Summary [file 41467_2022_30059_MOESM3_ESM.pdf]

Corresponding author(s): Kenneth R Cohen

Last updated by author(s): 2023-03-24

Reporting Summary

Nature Portfolio wishes to improve the reproducibility of the work that we publish. This form provides structure for consistency and transparency in reporting. For further information on Nature Portfolio policies, see our Editorial Policies and the Editorial Policy Checklist.

Statistics

For all statistical analyses, confirm that the following items are present in the figure legend, table legend, main text, or Methods section.

n/a	Confirmed	
	Y	The exact sample size (*n*) for each experimental group/condition, given as a discrete number and unit of measurement
	Y	A statement on whether measurements were taken from distinct samples or whether the same sample was measured repeatedly
	Y	The statistical test(s) used AND whether they are one- or two-sided<br>*Only common tests should be described solely by name; describe more complex techniques in the Methods section.*
	Y	A description of all covariates tested
	Y	A description of any assumptions or corrections, such as tests of normality and adjustment for multiple comparisons
	Y	A full description of the statistical parameters including central tendency (e.g. means) or other basic estimates (e.g. regression coefficient) AND variation (e.g. standard deviation) or associated estimates of uncertainty (e.g. confidence intervals)
	Y	For null hypothesis testing, the test statistic (e.g. *F*, *t*, *r*) with confidence intervals, effect sizes, degrees of freedom and *P* value noted<br>*Give P values as exact values whenever suitable.*
NA		For Bayesian analysis, information on the choice of priors and Markov chain Monte Carlo settings
NA		For hierarchical and complex designs, identification of the appropriate level for tests and full reporting of outcomes
	Y	Estimates of effect sizes (e.g. Cohen's *d*, Pearson's *r*), indicating how they were calculated

Our web collection on statistics for biologists contains articles on many of the points above.

Software and code

Policy information about availability of computer code

Data collection	SQL used to analyrt analyrical data from administrative claims database.
Data analysis	R3.63 version was used to analyze the data. Pertaining codes are provided.

For manuscripts utilizing custom algorithms or software that are central to the research but not yet described in published literature, software must be made available to editors and reviewers. We strongly encourage code deposition in a community repository (e.g. GitHub). See the Nature Portfolio guidelines for submitting code & software for further information.

Data

Policy information about availability of data

All manuscripts must include a data availability statement. This statement should provide the following information, where applicable:

- Accession codes, unique identifiers, or web links for publicly available datasets
- A description of any restrictions on data availability
- For clinical datasets or third party data, please ensure that the statement adheres to our policy

Description of analytical datasets is provided in Fig 1. No publicaly available dataset is used.
